# Network topology of NaV1.7 mutations in sodium channel-related painful disorders

**DOI:** 10.1186/s12918-016-0382-0

**Published:** 2017-02-24

**Authors:** Dimos Kapetis, Jenny Sassone, Yang Yang, Barbara Galbardi, Markos N. Xenakis, Ronald L. Westra, Radek Szklarczyk, Patrick Lindsey, Catharina G. Faber, Monique Gerrits, Ingemar S. J. Merkies, Sulayman D. Dib-Hajj, Massimo Mantegazza, Stephen G. Waxman, Giuseppe Lauria, on behalf of the PROPANE Study Group, Michela Taiana, Margherita Marchi, Raffaella Lombardi, Daniele Cazzato, Filippo Martinelli Boneschi, Andrea Zauli, Ferdinando Clarelli, Silvia Santoro, Ignazio Lopez, Angelo Quattrini, Janneke Hoeijmakers, Maurice Sopacua, Bianca de Greef, Hubertus Julius Maria Smeets, Rowida Al Momani, Jo Michel Vanoevelen, Ivo Eijkenboom, Sandrine Cestèle, Oana Chever, Rayaz Malik, Mitra Tavakoli, Dan Ziegler

**Affiliations:** 1 Bioinformatics Unit, IRCCS Foundation “Carlo Besta” Neurological Institute, Milan, Italy; 2 Neuroalgology Unit, IRCCS Foundation “Carlo Besta” Neurological Institute, Milan, Italy; 3 Department of Neurology, Yale University School of Medicine, New Haven, USA; 4 Center for Neuroscience and Regeneration Research, Yale University School of Medicine, New Haven, USA; 5 Department of Clinical Genetics, Maastricht University Medical Center, Maastricht, The Netherlands; 6 Department of Knowledge Engineering, Maastricht University, Maastricht, The Netherlands; 7 Department of Neurology, Maastricht University Medical Center, Maastricht, The Netherlands; 8 Department of Neurology, Spaarne Hospital, Hoofddorp, The Netherlands; 9 Laboratory of Excellence Ion Channel Science and Therapeutics, Institute of Molecular and Cellular Pharmacology, CNRS UMR7275 & University of Nice-Sophia Antipolis, Valbonne, France; 10 Present address: San Raffaele Scientific Institute and Vita-Salute University, Milan, Italy

**Keywords:** Sodium channel, Neuropathic pain, Structural modeling, Network analysis

## Abstract

**Background:**

Gain-of-function mutations in *SCN9A* gene that encodes the voltage-gated sodium channel NaV1.7 have been associated with a wide spectrum of painful syndromes in humans including inherited erythromelalgia, paroxysmal extreme pain disorder and small fibre neuropathy. These mutations change the biophysical properties of NaV1.7 channels leading to hyperexcitability of dorsal root ganglion nociceptors and pain symptoms. There is a need for better understanding of how gain-of-function mutations alter the atomic structure of Nav1.7.

**Results:**

We used homology modeling to build an atomic model of NaV1.7 and a network-based theoretical approach, which can predict interatomic interactions and connectivity arrangements, to investigate how pain-related NaV1.7 mutations may alter specific interatomic bonds and cause connectivity rearrangement, compared to benign variants and polymorphisms. For each amino acid substitution, we calculated the topological parameters betweenness centrality (B_*ct*_), degree (D), clustering coefficient (CC_*ct*_), closeness (C_*ct*_), and eccentricity (E_*ct*_), and calculated their variation (Δ_*value*_ = mutant _*value*_-WT _*value*_). Pathogenic NaV1.7 mutations showed significantly higher variation of |ΔB_*ct*_| compared to benign variants and polymorphisms. Using the cut-off value ±0.26 calculated by receiver operating curve analysis, we found that ΔB_*ct*_ correctly differentiated pathogenic NaV1.7 mutations from variants not causing biophysical abnormalities (nABN) and homologous SNPs (hSNPs) with 76% sensitivity and 83% specificity.

**Conclusions:**

Our *in-silico* analyses predict that pain-related pathogenic NaV1.7 mutations may affect the network topological properties of the protein and suggest |ΔB_*ct*_| value as a potential *in-silico* marker.

**Electronic supplementary material:**

The online version of this article (doi:10.1186/s12918-016-0382-0) contains supplementary material, which is available to authorized users.

## Background

*SCN9A* gene encodes the alpha-subunit of voltage-gated sodium channel NaV1.7 that is expressed in dorsal root ganglion (DRG) nociceptors and in sympathetic neurons. NaV1.7 is folded into four homologous domains, each containing six transmembrane helices (S1-S6). S1–S4 helices form the voltage-sensing domain (VSD) and highly conserved basic residues in S4 sense the electric field across the membrane. S5–S6 helices with the re-entrant extracellular loop in between form the pore domain (PD) [1]. Membrane depolarisation induces a conformational change in the VSD that, through the S4-S5 linker, is transmitted to the PD and prompt the gate to open, allowing the passage of sodium ions through the pore [2]. Opening and closing of the channel modulate the subthreshold membrane potential of nociceptors and play a key role in regulating their firing.

Missense mutations in *SCN9A* have been associated to a spectrum of painful conditions in humans [3], including inherited erythromelalgia (IEM), [4–13], paroxysmal extreme pain disorder (PEPD) [14–17], and small fibre neuropathy (SFN) [18, 19]. Voltage-clamp recording, performed in transfected cell lines and DRG neurons in vitro, showed that IEM-related mutations enhance the activation of NaV1.7 through a hyperpolarising shift and a slower deactivation that keeps the channel open longer once it is activated [3], thus generating a larger-than-normal inward sodium current, with greater biophysical changes at higher temperature [20]. PEPD-related NaV1.7 mutations impair channel inactivation and prolong action potentials and repetitive nociceptor firing in response to provoking stimuli, such as stretching and exposure to cold temperatures [14, 16, 21]. NaV1.7 mutations identified in SFN patients display a spectrum of electrophysiological signatures, including impaired slow inactivation, depolarised slow and fast inactivation and enhanced resurgent currents [18].

Overall, all the disease-related NaV1.7 mutations are pro-excitatory for the NaV1.7 channel, thus increasing nociceptor excitability. For those NaV1.7 mutations that have been studied by structural modelling, the gain-of-function effect stems from functionally significant changes in the biomolecular structure of NaV1.7 channel [22–24]. Accordingly, gain-of-function mutations found in IEM, PEPD, and SFN patients might be expected to produce functionally significant changes in the protein structure of NaV1.7, whereas single nucleotide polymorphisms (SNPs) or variants not associated with disease would not be expected to modify the NaV1.7 protein structure in functionally significant ways. Previous NaV1.7 structural modelling, combined with functional studies, showed that the disruption of the hydrophobic ring by the F1449V [24] or the in-frame deletion Leu955Del [22] contribute to destabilizing the NaV1.7 closed-state. These studies suggest that homology modelling is a useful tool to predict functional changes in the biomolecular structure of Nav1.7. However, the nature and extent of interatomic bond variations in NaV1.7 protein structure caused by amino acid changes have not been examined over a spectrum of mutations and SNPs.

Structural modelling combined with network theory has been widely exploited in studying protein structure to identify the emergent features of global connectivity. Indeed, several studies have used network theory to provide important insights in the local topology of interactions from a global prospective with examples from the field of allosteric communication pathways [25], protein-protein interactions [26], catalytic site residues in enzymes [27] and protein-folding mechanisms [28]. Several methods have been proposed in the literature to transform the protein structures into a network by considering: (a) the C-alpha/C-beta atoms in the amino acid residues, as in a protein backbone network [29] (b) description of the atomic contacts between residues that also feature correlated motions [30–32] or (c) weak and strong non-covalent protein structure network considering atom-atom interaction at the side chain level which has been proven to provide valuable biological insights [30, 33, 34]. These studies have shown that network analysis of a protein can yield a useful method to characterize the topology of the constituent amino acid residues. Protein topologies and interaction connectivity could often produce distinct small-world networks proprieties [28, 35, 36], thus having high local connectivity of residue nodes with a smaller number of long-range residue-residue interactions.

In the present study, we aimed at elucidating specific interatomic bond variations caused by amino acid changes in NaV1.7 structure by using a network-based method. We tested the hypothesis that mutations associated with IEM, PEPD and SFN cause specific types of interatomic bonds variation of NaV1.7 that can be quantified by a network-based theoretical approach able to reduce the complexity of the three-dimensional protein architecture to one-dimensional graphs [28].

## Methods

### Protocol description

**Fig. 1:**
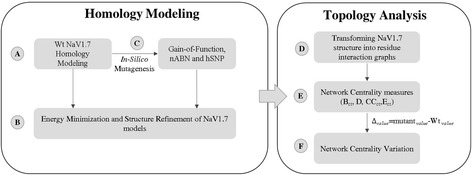
NaV1.7 computational protocol overview. *A* NaV1.7 WT homology modelling of based on the bacterial NavAb sodium channel template. *B* Energy minimization and structure refinement of the protein structure with YAMBER force field and FG-MD server. *C* *In-silico* mutagenesis for pathogenetic and control group (nABN/hSNPs) mutations. *D* Transforming NaV1.7 structure into residue interaction graphs. The construction of inter-residue network was based on interatomic bonds (hydrophobic, hydrogen bonds, salt-bridges, cation-π and π-π stacking interactions) using the commands “ListIntAtom” and “ListIntBo” via YASARA software. The *de novo* network construction for each mutant and WT models is achieved considering the predicted binary interatomic bonds. *E*-*F*. Network centrality calculation and their relative variation between mutant and WT (Δ_*value*_ = mutant _*value*_-WT _*value*_)

The overall method is summarized in Fig. 1. Our methodology can be encapsulated in a protocol that has two main components: homology modelling and topology analysis. The main steps of the current protocol are: (A) Homology modelling of NaV1.7 WT based on the bacterial NavAb sodium channel template. (B) Energy minimization and structure refinement of the protein structure (C) *In-silico* mutagenesis is performed for pathogenetic and control group (nABN/hSNPs) mutations (Table 1). (D) Construction of inter-residue network based on weak and strong noncovalent interactions (E) Network centrality calculation and (F) the difference between mutated and WT mutated (Δ_*value*_ = mutant _*value*_-WT _*value*_).

**Table 1:** ΔB_*ct*_ values of NaV1.7 mutations associated to IEM, SFN and PEPD

Disease	Mutation	Amino acid Properties	Channel Part	ΔB_*ct*_	Reference
IEM	I136V	=H_*Φ*_O	VSD (S1;D_I_)	0.12	[12, 58, 69]
	S211P	Polar → H_*Φ*_O	VSD (S4;D_I_)	-1.09	[70]
	F216S	H_*Φ*_O → polar	VSD (S4;D_I_)	-1.71	[11, 57]
	L823R	H_*Φ*_O → charged	VSD (S4;D_II_)	1.23	[7, 71]
	W1538R	H_*Φ*_O → charged	VSD (S2-S3;D_IV_)	0.18	[72]
	I234T	H_*Φ*_O → polar	S4-S5 (D_I_)	2.33	[73]
	S241T	=Polar	S4-S5 (D_I_)	0.34	[23, 74, 75]
	I848T	H_*Φ*_O → polar	S4-S5 (D_II_)	-5.83	[4, 9, 17, 47, 59]
	L858H	H_*Φ*_O → charged	S4-S5 (D_II_)	-1.85	[4, 9, 17]
	L858F	=H_*Φ*_O	S4-S5 (D_II_)	-1.74	[5, 11, 76]
	G856D^a^	H_*Φ*_O → charged	S4-S5 (D_II_)	-0.55	[19]
	A863P	=H_*Φ*_O	S4-S5 (D_II_)	-0.32	[6]
	P1308L	=H_*Φ*_O	S4-S5 (D_III_)	0.04	[21]
	V1316A	=H_*Φ*_O	S4-S5 (DI_III_)	0.36	[47, 77]
	A1632E^b^	H_*Φ*_O → charged	S4-S5 (D_IV_)	0.27	[78]
	N395K	polar → charged	Pore (S6;D_I_)	5.32	[11, 79]
	V400M	=H_*Φ*_O	Pore (S6;D_I_)	-0.68	[23, 80]
	V872G	=H_*Φ*_O	Pore (S5;D_I_)	-2.48	[81]
	F1449V	=H_*Φ*_O	Pore (S6;D_III_)	-0.51	[10, 23]
	A1746G	=H_*Φ*_O	Pore (S6;D_IV_)	1.40	[72]
SFN	R185H	=charged	VSD (S2-S3;D_I_)	0	[18]
	I228M	=H_*Φ*_O	VSD (S4;D_I_)	2.04	[18, 82]
	I739V	=H_*Φ*_O	VSD (S1;D_II_)	0.54	[18, 83]
	M1532I	=H_*Φ*_O	VSD (S2-S3;D_IV_)	0.15	[18]
	M932L	=H_*Φ*_O	Loop Pore (D_II_)	0.46	[18]
PEPD	V1298D	H_*Φ*_O → charged	S4-S5 (D_III_)	-0.81	[14]
	V1298F	=H_*Φ*_O	S4-S5 (D_III_)	-0.004	[14, 15, 21]
	V1299F	=H_*Φ*_O	S4-S5 (D_III_)	0.07	[14, 15, 17]
	G1607R	H_*Φ*_O → charged	S4-S5 (D_III_)	0.62	[84]
	M1627K	H_*Φ*_O → charged	S4-S5 (D_IV_)	1.22	[14, 16, 17, 85]

*IEM* Inherited erythromelalgia, *SFN* Small Fibre Neuropathy, *PEPD* Paroxysmal extreme pain disorder, *nABN* no biophysical abnormalities, *H*_*Φ*_ *O* Hydrophobic. ΔB_*ct*_ was calculated as (mutated B_*ct*_ – Wild-type B_*ct*_) × 100

^a^This mutation associates with clinical features of IEM and SFN

^b^This mutation causes symptoms common both to IEM and PEPD

#### NaV1.7 homology modelling

A homology model of the closed-state pore domain of the NaV1.7 was generated using the crystal structure of the bacterial *Arcobacter bultzeri* NaV channel NaVAb [37] as a template with the human sequence NM_002977.3 through the MEMOIR server [38]*.* Gap region (269-340, D_I_) between template-target alignment and interdomain loop regions (416-726, D_I_-D_II_; 967-1175, D_II_-D_III_; 1458-1498, D_III_-D_IV_) were excluded from *in-silico* mutagenesis (Fig. 1A). The NaVAb template shared 28% sequence identity for D_I_, 24% for D_II,_ 28% for D_III_ and 28% for D_IV_ (overall 27% sequence identity). The four homologous domains were modelled in the clockwise direction viewed from the extracellular side as previously suggested [39, 40]*.* Ab-initio modelling was performed to extend the S6 helices of the PD using the Iterative Threading ASSEmbly Refinement (I-TASSER) server [41]. The final model was subjected to energy minimization and model refinement using the YAMBER force field [42] and the Fragment-Guided Molecular Dynamics (FG-MD) server [43]. The NaV1.7 WT model was subjected to stereochemical analysis with RAMPAGE server (http://services.mbi.ucla.edu/). RAMPAGE provides results in a graphical form that shows the number of residues falling in favoured region, allowed region and in outlier region.

#### In-Silico Mutagenesis of NaV1.7 pathogenetic and control mutations

We performed *in-silico* mutagenesis via WT domain replacement of NaV1.7 mutations found in IEM, PEPD or SFN patients in which gain-of-function was demonstrated by cell electrophysiology assay and that do not alter the biophysical properties of the channel (nABN). To increase the number of control variants, we added missense SNPs identified between *SCN9a* homologous genes sharing >90% nucleotide sequence identity using the NCBI HomoloGene Database [44]. We constructed the phylogenetic tree of the multiple sequence alignment using ClustalW via neighbor joining method (Additional file 1: S1 Text; https://www.ebi.ac.uk/Tools/phylogeny/clustalw2_phylogeny/). The mutated models were further subjected to energy minimization and model refinement using the YAMBER force field [42] and the FG-MD server (Fig. 1B) [43]. Such hSNPs have previously been used in similar studies [45–47]. All the mutations and SNPs are reported in Additional file 2: Table S1.

#### Transforming NaV1.7 structure into residue interaction graphs

NaV1.7 structures were transformed into mathematical graphs by identifying interatomic bonds between the amino acids. The amino acid residues form the nodes and inter-node contact interaction form the edges of the graph (Fig. 1D). We identified the interatomic bonds (hydrophobic, hydrogen bonds, salt-bridges, cation-π and π-π stacking interactions) between two residues *i* and *j* as long as the atom-atom distance between them was less than 5.0 Å using the commands “ListIntAtom” and “ListIntBo” via YASARA software (Yet Another Scientific Artificial Reality Application, www.yasara.org). Hydrophobic contacts between residues were considered in the following atom groups: (a) the first carbon of CH_3_-, -CH_2_- and CHC_3_ (b) sp^2^ carbons (phenolic rings). π-π stacking were considered between (a) sp^2^ carbons with a hydrogen and (b) carbon, nitrogen, oxygen or sulphur atoms in planar phenolic rings. Cation-π formation was considered to be a π-π contact with the difference being that one of the interaction partners is a cation. The *de novo* network construction for each mutant and WT models is achieved considering the predicted binary interatomic bonds identified through YASARA software.

#### Topological metrics and network visualization

We computed some of the most well-known network centrality measures for each mutant and WT network NaV1.7 graph using the Cytoscape plugin NetworkAnalyzer [48], namely:

Betweenness Centrality (B_*ct*_) and edge Betweenness centrality (EB_*ct*_): B_*ct*_ [49] is defined as the fraction of shortest pathways between all pairs of nodes of the network that go through that node. Let G = (N, E) a graph, where N is the set of the nodes and E is the set of the edges. For each node n and m in N, let d (n, m) the distance between n and m. We define

1 $$ \mathrm{Betweenness}\ \mathrm{centrality}\ \left(\mathrm{n}\right)={\displaystyle {\sum}_{\mathrm{s}\ \ne \mathrm{n}\ \ne \mathrm{t}}}\frac{\upsigma_{\mathrm{s}\mathrm{t}\ }\left(\mathrm{n}\right)}{\upsigma_{\mathrm{s}\mathrm{t}}}, $$

where s, t ∈N, σ_st_ (n) is the number of shortest paths from s to t that n lies on, and σ_st_ denotes the number of shortest paths from s to t. It accounts the importance of a node facilitating interactions between other nodes. For example, a node with high Bct can operate as a bridge on many shortest paths between other nodes in the network. It is a measure of how powerful a node is able to transfer (high B_*ct*_) or interrupt (low B_*ct*_) the spread of information on the fastest connection between two nodes. Similarly, the EB_*ct*_ of an edge is the number of shortest paths between pairs of nodes that run along it. We define:

2 $$ \mathrm{Edge}\ \mathrm{Betweenness}\ \left(\mathrm{e}\right)={\displaystyle {\sum}_{{\mathrm{n}}_{\mathrm{i}}\ \in\ \mathrm{N}}}{\displaystyle {\sum}_{{\mathrm{n}}_{\mathrm{j}}\kern0.5em \in\ \mathrm{N}\backslash \left\{{\mathrm{n}}_{\mathrm{i}}\right\}\ }}{\displaystyle \sum }\ \frac{{\upsigma_{{\mathrm{n}}_{\mathrm{i}}}}_{{\mathrm{n}}_{\mathrm{j}}}\ \left(\mathrm{e}\right)\ }{{\upsigma_{{\mathrm{n}}_{\mathrm{i}}}}_{{\mathrm{n}}_{\mathrm{j}}}}, $$

Where *N* = set of nodes; *E* = set of edges; ${\upsigma_{{\mathrm{n}}_{\mathrm{i}}}}_{{\mathrm{n}}_{\mathrm{j}}}$ = number of shortest paths between n_i_ and n_j_; ${\upsigma_{{\mathrm{n}}_{\mathrm{i}}}}_{{\mathrm{n}}_{\mathrm{j}}}\ \left(\mathrm{e}\right)$ = number of shortest paths between n_i_ and n_j_ which pass through e ∊ E;

Degree (D): D [49] of a node (k) is defined as the total number of nodes that it is directly connected to;

Clustering Coefficient (CC_*ct*_): Clustering Coefficient [49] is a metric commonly employed to identify well-connected sub-components in network which represents the interconnectivity of neighbors of the node. It measures the degree to which nodes tend to cluster together and is defined as the fraction of triangles around a node among the total number of possible triangles. We define

3 $$ \mathrm{Clustering}\ \mathrm{Coefficient}\ \left(\mathrm{n}\right)=\frac{2{\mathrm{e}}_{\mathrm{n}}}{{\mathrm{k}}_{\mathrm{n}\ }\left({\mathrm{k}}_{\mathrm{n}} - 1\right)}, $$

where k_n_ is the number of neighbors of n and e_n_ is the number of connected pairs between all neighbors of n;

Closeness centrality (C_*ct*_): Cct is defined as the sum of the inverted distances, i.e. farness, to all other nodes in the graph. It captures the basic intuition that the closer a node is to all other nodes in terms of path length, the more important it is. Mathematically, C_*ct*_ of a node n is defined as the inverse of the sum of shortest paths from n to all other nodes m in network. We define

4 $$ \mathrm{Closeness}\ \left(\mathrm{n}\right)=\frac{1}{\mathrm{average}\ \left(\mathrm{d}\left(\mathrm{n},\ \mathrm{m}\right)\right)} $$

Eccentricity (E_*ct*_): Ect measures the distance between a node n and the most distance node m; if the E_*ct*_ of the node n is low, this means that all other nodes are in proximity whereas a high E_*ct*_ means that there is at least one node (and all its neighbors) that is far from node n. We define E_*ct*_ maximum non-infinite length of a shortest path between n and another node in the network. We define

5 $$ \mathrm{Eccentricity}\ \left(\mathrm{n}\right)= \max \left\{\mathrm{d}\left(\mathrm{n},\ \mathrm{m}\right)\ :\ \mathrm{m}\kern0.5em \in \kern0.5em \mathrm{N}\right\} $$

#### Network centrality measure variation

For each network centrality measures we calculated the difference between mutant and WT values defined as Δvalue (Δvalue = mutant value – WT value). The NaV1.7 amino acid network was visualized using Cytoscape’s Organic layout, which is a force-directed layout algorithm similar to the Fruchterman-Reingold approach [50].

### Statistical analysis

Statistical analyses were performed using the R statistical Package [51]. Data are indicated as mean ± SD. Statistical significance was determined by the Wilcoxon signed-ranked test (*p* <0.05). The receiver operating characteristics (ROC) curve was used to assess the discriminatory power of centrality measure variations between pathogenetic NaV1.7 mutations and control groups (nABN and hSNPs). The upper-angle of ROC corresponding to the best sensitivity and specificity was used to identify the best cut-off value.

## Results

### NaV1.7 interatomic structure graph design

**Fig. 2:**
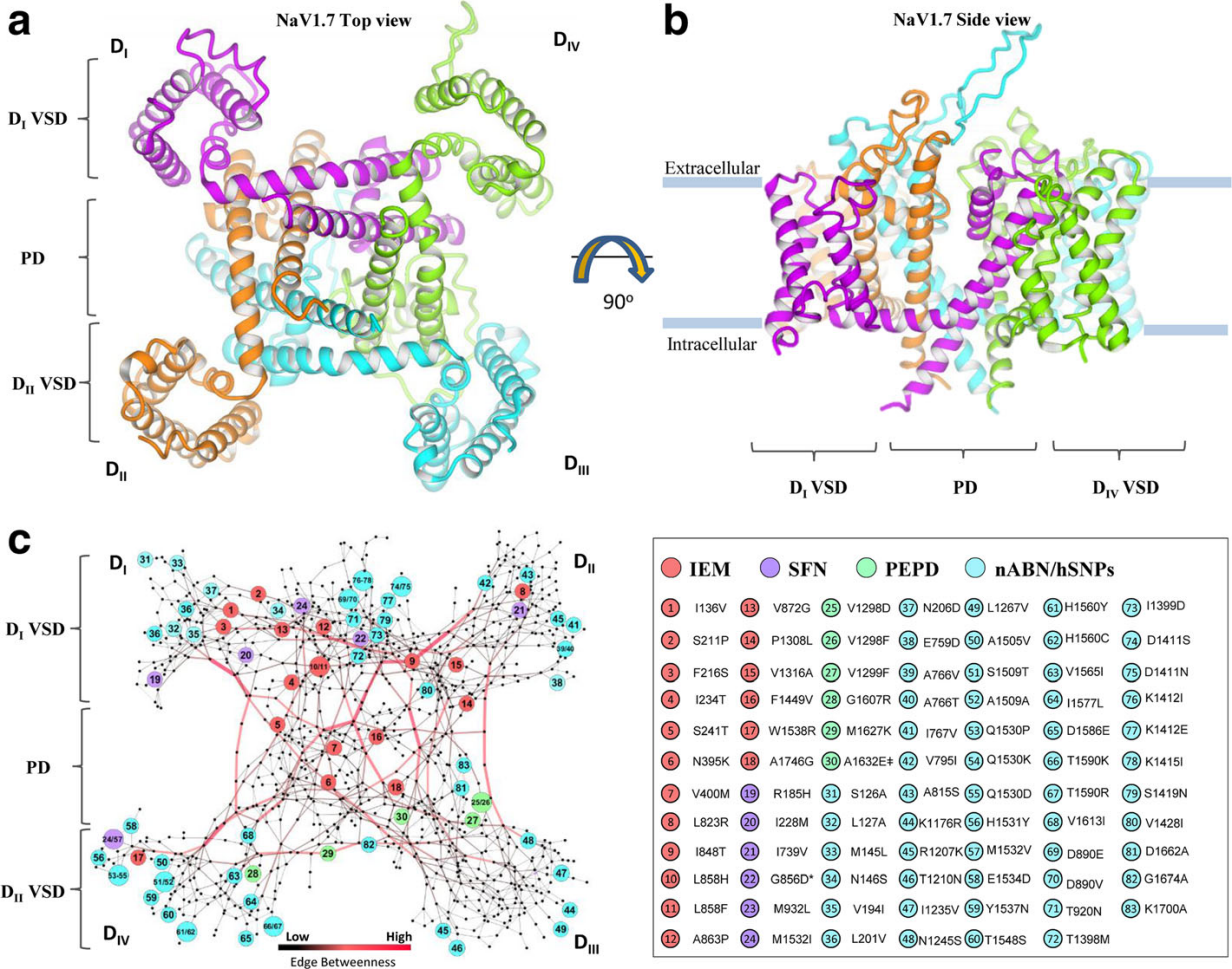
NaV1.7 structure and inter-atomic network features. **a** View of the sodium channel α-subunit from the intracellular side of the membrane NaV1.7 is folded into four repeated domains (D_I_–D_IV_); helices S1–S4 comprise the voltage-sensing domain (VSD); helices S5–S6 and their intracellular linker comprise the pore domain (PD). **b** Intramembrane view of the folded model of NaV1.7. **c** The graph shows the topology of the mutations found in patients with inherited erythromelalgia (IEM; *red*), paroxysmal extreme pain disorder (PEPD; *green*), small-fibre neuropathy (SFN; *purple*) and the amino acid substitution with no biophysical abnormalities (nABN) and homologous SNPs (*light blue*). Nodes represent the residues and edges of the interatomic bonds. *Red* and *black* edges represent high (*red*) or low (*grey*) edge betweenness centrality (EB_*ct*_) values, respectively. Edge thickness are proportional to EB_*ct*_ and reveal that a high number of shortest paths pass through few edges. *This mutation associates with clinical features of IEM and SFN. ǂThis mutation causes in vitro biophysics changes and in vivo symptoms common both to IEM and PEPD. The NaV1.7 amino acid network were visualized using Cytoscape’s Organic layout, which is a force-directed layout algorithm similar to the Fruchterman-Reingold approach

We performed homology modelling to construct the tertiary structure of the closed-state NaV1.7 sodium channel (Fig. 1). We constructed the atomic model of NaV1.7 sodium channel using the MEMOIR server [38] based on the crystal structure of the bacterial *Arcobacter bultzeri* NaV channel NaVAb as a template with the human sequence NM_002977.3*.* The first four helices S1–S4 form the VSD and the last two helices S5–S6 form the PD (Fig. 2a and b). Gap region (269-340, S5-6 extracellular linker in D_I_) between template-target alignment and interdomain loop regions (416-726, D_I_-D_II_; 967-1175, D_II_-D_III_; 1458-1498, D_III_-D_IV_) were excluded from *in-silico* mutagenesis. The four homologous domains were modelled in the clockwise direction viewed from the extracellular side as suggested previously [39, 40]*.* *Ab-initio* modelling was performed to extend the S6 helices of the PD using the Iterative Threading ASSEmbly Refinement (I-TASSER) server [41]. The final model was subjected to energy minimization and model refinement using the YAMBER force field [42] and the Fragment-Guided Molecular Dynamics (FG-MD) server [43] (Additional file 3: NaV1.7 pdb file). The RAMPAGE results for the NaV1.7 model showed 88.5% residues in most favored region (Additional file 4: Figure S1), 9% (90 residues) in allowed region and 2.5% (25 residues) in outlier region. A good quality Ramachandran plot has over 90% residues in the most favoured regions [52] therefore Ramachandran plot of NaV.17 it is close to a good quality model (88.5% residues in most favoured regions).

We performed *in-silico* mutagenesis for 18 mutations causing IEM, 6 mutations causing SFN, 6 mutations causing PEPD (Additional file 2: Table S1), 4 mutations not causing biophysical abnormalities (nABN) in the channel (N1245S: [53]; L1267V: [53]; V1428I and T920N: Waxman, Dib-Hajj and Mantegazza, unpublished observations) and 49 SNPs identified among human and homologous mammalian (hSNPs) *SCN9A* genes with >90% sequence identity (Additional file 5: S2 Text). All the disease-related mutations had previously been characterized by electrophysiological assays, and found to confer gain-of-function changes to the NaV1.7 channel (Additional file 2: Table S1). The WT and mutant NaV1.7 structures were transformed into undirected graphs by the identification of hydrophobic, cation-π and π-π stacking interactions and hydrogen bonds (H-bonds) among the amino acids. In the resulting graph, amino acids are the nodes and their interactions are the edges (Fig. 2c).

### Analyses of the interatomic variations caused by gain-of-function mutations

Previous studies showed that gain-of-function mutations change the biophysical properties of the channel NaV1.7 [4–9, 14–16, 18, 54] but the underlying interatomic variations are yet to be investigated. We analyzed the interatomic variations by calculating the network centrality parameters (B_*ct*_, D, CC_*ct*_, C_*ct*_, E_*ct*_; see methods for detailed definitions) of WT and mutated residues and the value of the variation (Δ_*value*_ = mutant _*value*_ - WT _*value*_, ΔB_*ct*_, ΔD, ΔCC_*ct*_, ΔC_*ct*_, ΔE_*ct*_) associated with each gain-of-function NaV1.7 mutation, nABN and hSNP. B_*ct*_ is a measure of the centrality of a node *n* defined as the fraction of shortest pathways between all pairs of nodes (*s*, *t*) of the network that go through that node *n* [49, 55]. D of a node *n* is defined as the total number of nodes that it is directly connected to [49, 55]. CC_*ct*_ is a metric commonly employed to identify well-connected sub-components in network which represents the interconnectivity of neighbors of a node *n* [35, 49]. C_*ct*_ is defined as the sum of the inverted distances of a node *n*, i.e. farness, to all other nodes in the graph. It captures the basic intuition that the closer a node is to all other nodes, the more important it is [56]. Eccentricity (E_*ct*_) of a node *n* is the greatest distance from a node *n* to any other node *m* [55].

**Fig. 3:**
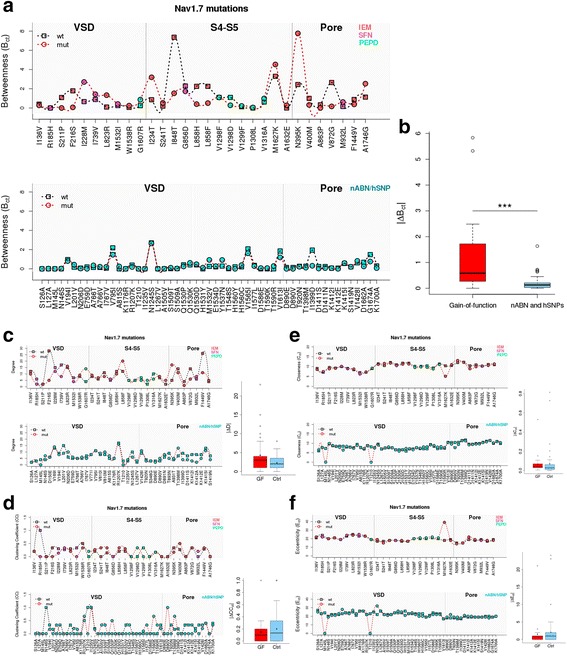
Topological parameter profiles of NaV1.7 gain-of-function mutations and nABN and hSNPs. **a** The *upper panel* shows the B_*ct*_ profile of gain-of-function mutation; the *lower panel* show the B_*ct*_ profile of nABN and hSNPs. Squares indicates B_*ct*_ values of WT amino acids, circles indicate B_*ct*_ values of mutated amino acids. The graphs highlight that the difference between B_*ct*_ value of mutated amino acids and B_*ct*_ value of WT amino acids is higher in the cohort of gain-of-function (GF) mutations (*upper panel*) compared to control (Ctrl) nABN and hSNPs (*lower panel*). B_*ct*_ values are multipled by 100. **b** The box plot shows the |ΔB_*ct*_| difference between gain-of-function mutations and the cohort of nABN and hSNP variants (mean gain-of-function |∆B_*ct*_| = 1.14 ± 1.40; nABN and hSNP ∆B_*ct*_ = 0.19 ± 0.28; ***p < 0.001 by Wilcoxon signed-ranked test). |∆B_*ct*_| values are multipled by 100; *dark horizontal lines* and the triangular symbol represent median and mean values respectively, with the box representing the 25th and 75th percentiles, the whiskers the 5th and 95th percentiles, and the dots the outliers. **c** The *upper panel* shows the Degree (D) profile of gain-of-function mutations; the *lower panel* show the D profile of nABN and hSNPs. Squares indicates D values of WT amino acids, *circles* indicate D values of mutated amino acids. The *box* plot shows the |ΔD| difference between gain-of-function (GF) mutations and the cohort of nABN and hSNP (Ctrl) variants (gain-of-function mean |∆D| = 4.3 ± 5.15; nABN and hSNP |∆D| = 2.37 ± 2.10; *p* > 0.05 by Wilcoxon signed-ranked test); *dark horizontal lines* and the triangular symbol represent median and mean values respectively, with the box representing the 25th and 75th percentiles, the whiskers the 5th and 95th percentiles, and the dots the outliers. **d** The *upper panel* shows the Clustering Coefficient (CC) profile of gain-of-function mutations; the *lower panel* show the CC profile of nABN and hSNPs. Squares indicates CC values of WT amino acids; circles indicate CC values of mutated amino acids. The box plot shows the |ΔCC_*ct*_| difference between gain-of-function (GF) mutations and the cohort of nABN and hSNP (Ctrl) variants (mean gain-of-function |∆CC_*ct*_| = 0.15 ± 0.20; nABN and hSNP |∆CC_*ct*_| = 0.20 ± 0.25; *p* > 0.05 by Wilcoxon signed-ranked test); *dark horizontal lines* and the triangular symbol represent median and mean values respectively, with the box representing the 25th and 75th percentiles, the whiskers the 5th and 95th percentiles, and the dots the outliers. **e** The *upper panel* shows the Closeness (C_ct_) profile of gain-of-function mutations; the *lower panel* show the C_ct_ profile of nABN and hSNPs. Squares indicates C_ct_ values of WT amino acids, *circles* indicate C_ct_ values of mutated amino acids. The *box* plot shows the ΔC_*ct*_ difference between gain-of-function (GF) mutations and the cohort of nABN and hSNP (Ctrl) variants (mean gain-of-function |∆C_*ct*_| = 0.6 ± 0.9; nABN and hSNP |∆C_*ct*_| = 0.7 ± 1.4; *p* > 0.05 by Wilcoxon signed-ranked test). C_*ct*_ and ∆C_*ct*_ values are multipled by 100; *dark horizontal lines* and the triangular symbol represent median and mean values respectively, with the box representing the 25th and 75th percentiles, the whiskers the 5th and 95th percentiles, and the dots the outliers. **f** The *upper panel* shows the Eccentricity (E_ct_) profile of gain-of-function mutations; the *lower panel* show the E_ct_ profile of nABN and hSNPs. Squares indicates E_ct_ values of WT amino acids, *circles* indicate E_ct_ values of mutated amino acids. The box plot shows the |ΔE_*ct*_| difference between gain-of-function (GF) mutations and the cohort of nABN and hSNP (Ctrl) variants (mean gain-of-function |∆E_*ct*_| = 1.53 ± 3.75; nABN and hSNP |∆E_*ct*_| = 2.05 ± 4.62; p > 0.05 by Wilcoxon signed-ranked test); *dark horizontal lines* and the triangular symbol represent median and mean values respectively, with the box representing the 25th and 75th percentiles, the whiskers the 5th and 95th percentiles, and the dots the outliers

Figure 3a-e show the profile of the topological parameters B_*ct*_, D, CC_*ct*_, C_*ct*_, and E_*ct*_ in WT and mutated residues. The graphs show that both gain-of-function mutations and nABN/hSNPs modify the D values (Fig. 3c and Additional file 6: Figure S2) and CC_*ct*_ values (Fig. 3d and Additional file 7: Figure S3) in a wide range but without significant differences between the groups (gain-of-function mean *∆*D = 4.30 ± 5.15; nABN and hSNP mean ∆D = 2.27 ± 2.1; p > 0.05 by Wilcoxon signed-ranked test; gain-of-function mean ∆CC_*ct*_ = 0.15 ± 0.20; nABN and hSNP mean ∆CC_*ct*_ = 0.20 ± 0.25; p > 0.05 by Wilcoxon signed-ranked test). Smaller variations were observed in C_*ct*_ values (Fig. 3e and Additional file 8: Figure S4) and E_*ct*_ values (Fig. 3f and Additional file 9: Figure S5) without significant differences between the groups (gain-of-function mean ∆C_*ct*_ = 0.65 ± 0.94; nABN and hSNP mean ∆C_*ct*_ = 0.71 ± 1.51; p > 0.05 by Wilcoxon signed-ranked test; gain-of-function mean ∆E_*ct*_ = 1.53 ± 3.75; nABN and hSNP mean ∆E_*ct*_ = 2.05 ± 4.62; p > 0.05 by Wilcoxon signed-ranked test). Overall, ΔD, ΔCC_*ct*_, ΔC_*ct*_, ΔE_*ct*_ did not differ significantly between gain-of-function mutations and nABN and hSNPs.

**Fig. 4:**
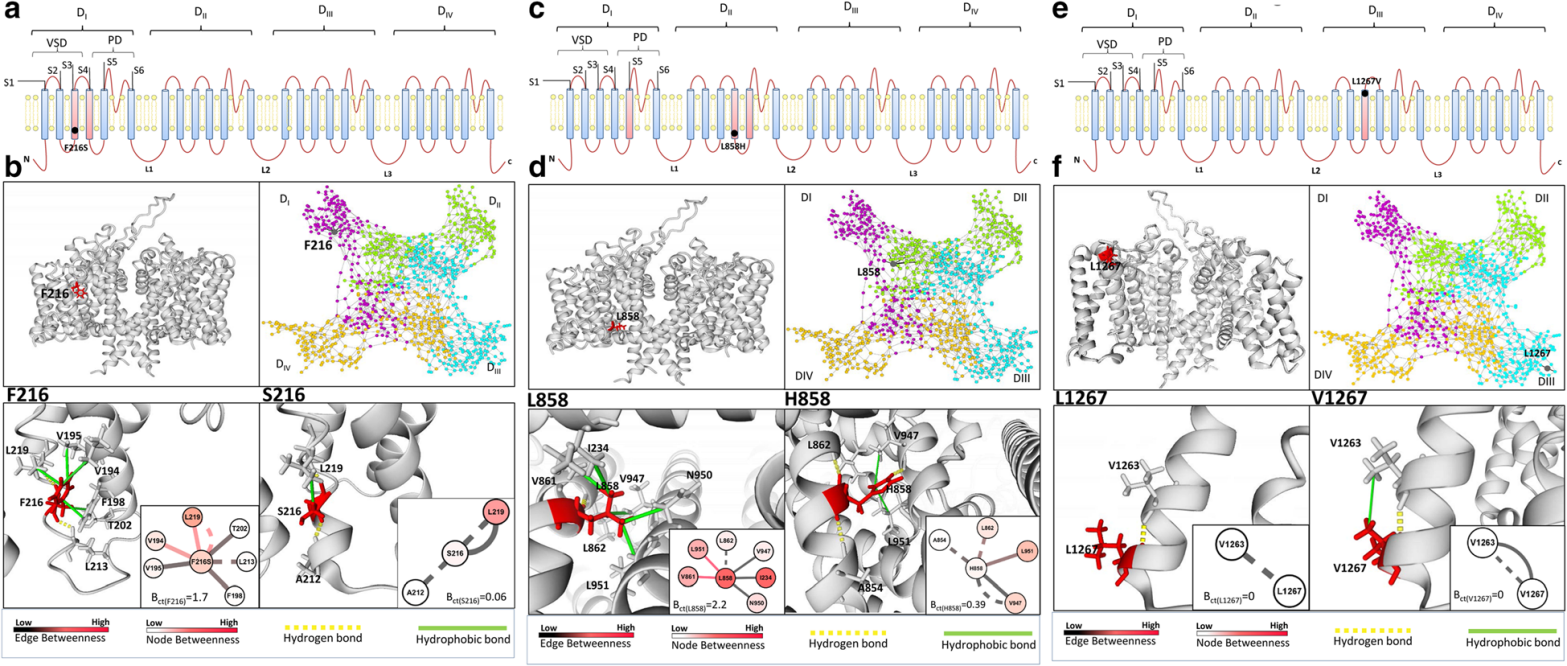
Structural modelling of NaV1.7 variants and their interatomic bonds. **a** The graph shows the NaV1.7 sodium channel topology and highlights the IEM associated mutation F216S and its intra-domain bond interaction (S3 and S4; depicted in *red*). **b** *Upper left* inset shows the intramembrane view of the NaV1.7 channel and the amino acid F216. *Upper right* inset shows network view of the four NaV1.7 channel domains (D_I_, *purple*; D_II_, *green*; D_III_, *light blue*; D_IV_, *orange*); the topology of amino acid F216 is showed as *grey* node. *Lower* insets show the bonds of WT amino acid F216 (*left*) and mutated amino acid S216 (*right*). Hydrophobic bonds are showed in green solid lines. H-bonds are showed with yellow dashed lines. F216 (D_I_, *red*) interacts with V194, V195, F198, T202 (S3, D_I_) and L219 (S4, D_I_) via hydrophobic bonds. F216 interact via H-bonds with L213 (formed by F216[NH] and L213[CO]) and L219 (F216[CO] with L219[NH]) located in S4, D_I_. The mutation F216S (*right*) interrupts all the hydrophobic interactions with S3 residues and created new H-bonds (S216[NH] with A212[CO]) causing a decrease of B_*ct*_ and EB_*ct*_ values. **c** The graph shows the NaV1.7 sodium channel topology and highlights the IEM associated mutation L858H and its inter-domain bond interaction (S4-S5 and S4; depicted in *red*). **d** *Upper left* inset shows the intramembrane view of the NaV1.7 channel and the amino acid L858. *Upper right* insets show network view of the four NaV1.7 channeldomains (D_I_, *purple*; D_II_, *green*; D_III_, *light blue*; D_IV_, *orange*); the topology of amino acid L858 is showed as *grey* node. *Lower* inset shows the bonds of WT amino acid L858 (*left*) and mutated amino acid H858 (*right*). Hydrophobic bonds are showed in *green solid lines*. H-bonds are indicated by *yellow dashed lines*. L858 residue (red, S4-S5; D_II_) interacts with I234 (D_I_; S4-S5), V861 (D_II_; S4-S5), N950, L951 and V947 (D_II_; S6) through hydrophobic bonds and through H-bonds with L862 (formed by L858[CO] and L862[NH]) located in D_II_; S4-S5. L858H mutation interrupts hydrophobic interaction with I234 (D_I_; S4-S5), V861 (D_II_; S4-S5), N950 and forms new H-bonds with A854 (formed by H858[NH] and A854[CO]) (D_II_; S4-S5) and V947 (formed by H858[NE2] and V947[CO]) (D_II_; S6). These changes decrease B_*ct*_ value of amino acid 858 from 2.2 to 0.39. **e** The graph shows the NaV1.7 sodium channel topology and highlights the nABN mutation L1267V that is located in the domain D_III_; S3 depicted in *red*. **f** *Upper left* inset show the intramembrane view of the NaV1.7 channel and the amino acid L1267. *Upper right* inset shows network view of the four NaV1.7 channel domains (D_I_, *purple*; D_II_, *green*; D_III_, *light blue*; D_IV_, *orange*); the topology of amino acid L1267 is showed as *grey* node. Lower inset show the bonds of WT amino acid L1267 (left) and mutated amino acid V1267 (*right*). Hydrophobic bonds are showed in *green solid lines*. H-bonds are indicated by *yellow dashed lines*. L1267[NH] (VSD in D_III_) interacts through H-bonds with V1263[CO]. V1267mutation interacts with V1263 through a hydrophobic bond. This change does not modify B_*ct*_ value of the residue 1267

We next analysed B_*ct*_ values and found that pathogenic NaV1.7 mutations are characterized by higher variations of ΔB_*ct*_ compared with non-pathogenic mutations and polymorphisms (Fig. 3a; Table 1 and 2). Indeed, |ΔB_*ct*_| was significantly higher in gain-of-function mutations compared with nABN and hSNPs (gain-of-function mean ∆B_*ct*_ = 1.14 ± 1.40; nABN and hSNP mean ∆B_*ct*_ = 0.19 ± 0.28; *p* < 0.001 by Wilcoxon signed-ranked test; Fig. 3b). ΔB_*ct*_ variations associated with Nav1.7 pathogenetic mutations and nABN variants are exemplified in the structural modeling shown in the Fig. 4.

**Table 2:** ΔB_*ct*_ values of NaV1.7 nABN and hSNPs

Type	Mutation	Amino acid properties	Channel Part	ΔB_*ct*_
hSNP	S126A	Polar → H_*Φ*_O	VSD (S1;D_I_)	0
	L127A	=H_*Φ*_O	VSD (S1;D_I_)	0.12
	M145L	=H_*Φ*_O	VSD (S1;D_I_)	0
	N146S	=Polar	VSD (S1;D_I_)	0.020
	V194I	=H_*Φ*_O	VSD (S3;D_I_)	-0.14
	L201V	=H_*Φ*_O	VSD (S2;D_I_)	0.259
	N206D	Polar → Charged	VSD (S2;D_I_)	-0.037
	E759D	=Charged	VSD (S1-S2;D_II_)	-0.18
	A766T	H_*Φ*_O → polar	VSD (S2;D_II_)	0.20
	A766V	=H_*Φ*_O	VSD (S2;D_II_)	0.06
	I767V	=H_*Φ*_O	VSD (S2;D_II_)	0.13
	V795I	=H_*Φ*_O	VSD (S3;D_II_)	-0.72
	A815S	H_*Φ*_O → polar	VSD (S3-S4;D_II_)	0.24
	K1176R	=Charged	VSD (S1;D_III_)	0.0004
	R1207K	=Charged	VSD (S1-S2;D_III_)	-0.19
	T1210N	=Polar	VSD (S1-S2;D_III_)	0
	I1235V	=H_*Φ*_O	VSD (S2;D_III_)	0
	A1505V	=H_*Φ*_O	VSD (S1;D_IV_)	0.00026
	S1509T	=Polar	VSD (S1;D_IV_)	0.092
	S1509A	Polar → H_*Φ*_O	VSD (S1;D_IV_)	0.0084
	Q1530P	Polar → H_*Φ*_O	VSD (S1-S2;D_IV_)	-0.166
	Q1530K	Polar → Charged	VSD (S1-S2;D_IV_)	0.20
	Q1530D	=Polar	VSD (S1-S2;D_IV_)	0.066
	H1531Y	Charged → Polar	VSD (S1-S2;D_IV_)	0.12
	M1532V	=H_*Φ*_O	VSD (S1-S2;D_IV_)	0.66
	E1534D	=Charged	VSD (S1-S2;D_IV_)	0.067
	Y1537N	=Polar	VSD (S1-S2;D_IV_)	0.63
	T1548S	=Polar	VSD (S2;D_IV_)	0.085
	H1560Y	Charged → Polar	VSD (S2-S3;D_IV_)	-0.20
	H1560C	=H_*Φ*_O	VSD (S2-S3;D_IV_)	0.17
	V1565I	=H_*Φ*_O	VSD (S2-S3;D_IV_)	-0.45
	I1577L	=H_*Φ*_O	VSD (S3;D_IV_)	-0.07
	D1586E	=Charged	VSD (S3;D_IV_)	0.07
	T1590K	Polar → Charged	VSD (S3-S4;D_IV_)	0.07
	T1590R	Polar → Charged	VSD (S3-S4;D_IV_)	0.29
	V1613I	=H_*Φ*_O	VSD (S4;D_IV_)	-0.67
nABN	N1245S^a^	=polar	VSD (S2-S3;D_III_)	-0.05
	L1267V^a^	=H_*Φ*_O	VSD (S3;D_III_)	0
	V1428I	=H_*Φ*_O	Pore (S6;D_III_)	0.19
	T920N	=Polar	Loop-P (D_II_)	0.04
hSNP	D890E	=Charged	Loop-P (D_II_)	-0.13
	D890V	Charged → H_*Φ*_O	Loop-P (D_II_)	-0.13
	T1398M	Polar → H_*Φ*_O	Loop-P (D_III_)	-0.14
	I1399D	H_*Φ*_O → Charged	Loop-P (D_III_)	-1.63
	D1411S	Charged → Polar	Loop-P (D_III_)	-0.20
	D1411N	Charged → Polar	Loop-P (D_III_)	-0.16
	K1412I	Charged → H_*Φ*_O	Loop-P (D_III_)	-0.0033
	K1412E	=Charged	Loop-P (D_III_)	-0.067
	K1415I	Charged → H_*Φ*_O	Loop-P (D_III_)	0.038
	S1419N	=Polar	Loop-P (D_III_)	0.001
	D1662A	Charged → H_*Φ*_O	Loop-P (D_IV_)	-0.43
	G1674A	=H_*Φ*_O	Loop-P (D_IV_)	-0.71
	K1700A	Charged → H_*Φ*_O	Loop-P (D_IV_)	-0.22

*hSNPs* homologous Single nucleotide Polymorphisms ΔB_*ct*_ was calculated as (mutated B_*ct*_ – Wild-type B_*ct*_) × 100

^a^Brouwer et al. [53]; H_*Φ*_O: Hydrophobic

Figure 4a and b shows the B_*ct*_ topological proprieties of the F216S mutation associated to IEM [11, 57]. In the WT protein, F216 is located in VSD (S4) of D_I_ and is predicted to mediates hydrophobic interactions with V194, V195, F198, T202 (S3, D_I_) and L219 (S4, D_I_). F216 is also predicted to mediate two H-bonds: F216[NH] with L213[CO] and F216[CO] with L219[NH] residues (S4, D_I_). Upon mutation, the hydrophobic interaction between F216S (S4) and the S3 residues (D_I_; VSD) are interrupted. The H-bonds F216[NH] with L213[CO] are interrupted. New H-bonds between S216[NH] and A212[CO] are created. All these changes yield negative B_*ct*_ variation (ΔB_*ct*_ = -1.71, Fig. 4a and b; Additional file 10: S1 YASARA; Additional file 11: S2 YASARA). L858H is another IEM-associated mutation [4, 9, 17]. In the WT protein, L858 is located in S4-S5 and is predicted to interacts with I234 (D_I_; S4-S5), V861 (D_II_; S4-S5), N950, L951 and V947 (D_II_; S6) through hydrophobic bonds and through H-bonds formed by L858[CO] and L862[NH]) (D_II_; S4-S5). L858H mutation interrupts hydrophobic interaction with I234 (D_I_; S4-S5), V861 (D_II_; S4-S5), N950 (D_II_; S6) and forms new H-bonds by H858[NH] and A854[CO] (D_II_; S4-S5) and by H858[CO] with V947[NH] (D_II_; S6) leading to a negative ΔB_*ct*_ value (-1.85) (Fig. 4c and d; Additional file 12: S3 YASARA; Additional file 13: S4 YASARA). L1267V is an example of nABN variant that is located in the VSD of D_III_ which is highly conserved between human and SCN9A homologous genes (Additional file 5: S2 Text). L1267 interacts with V1263 through H-bonds formed by L1267[NH] and V1263[CO]. Upon mutation, V1267 forms new hydrophobic bond with V1263 which does not cause B_*ct*_ variation (ΔB_*ct*_ =0) (Fig. 4e and f; Additional file 14:S5 YASARA; Additional file 15:S6 YASARA).

**Fig. 5:**
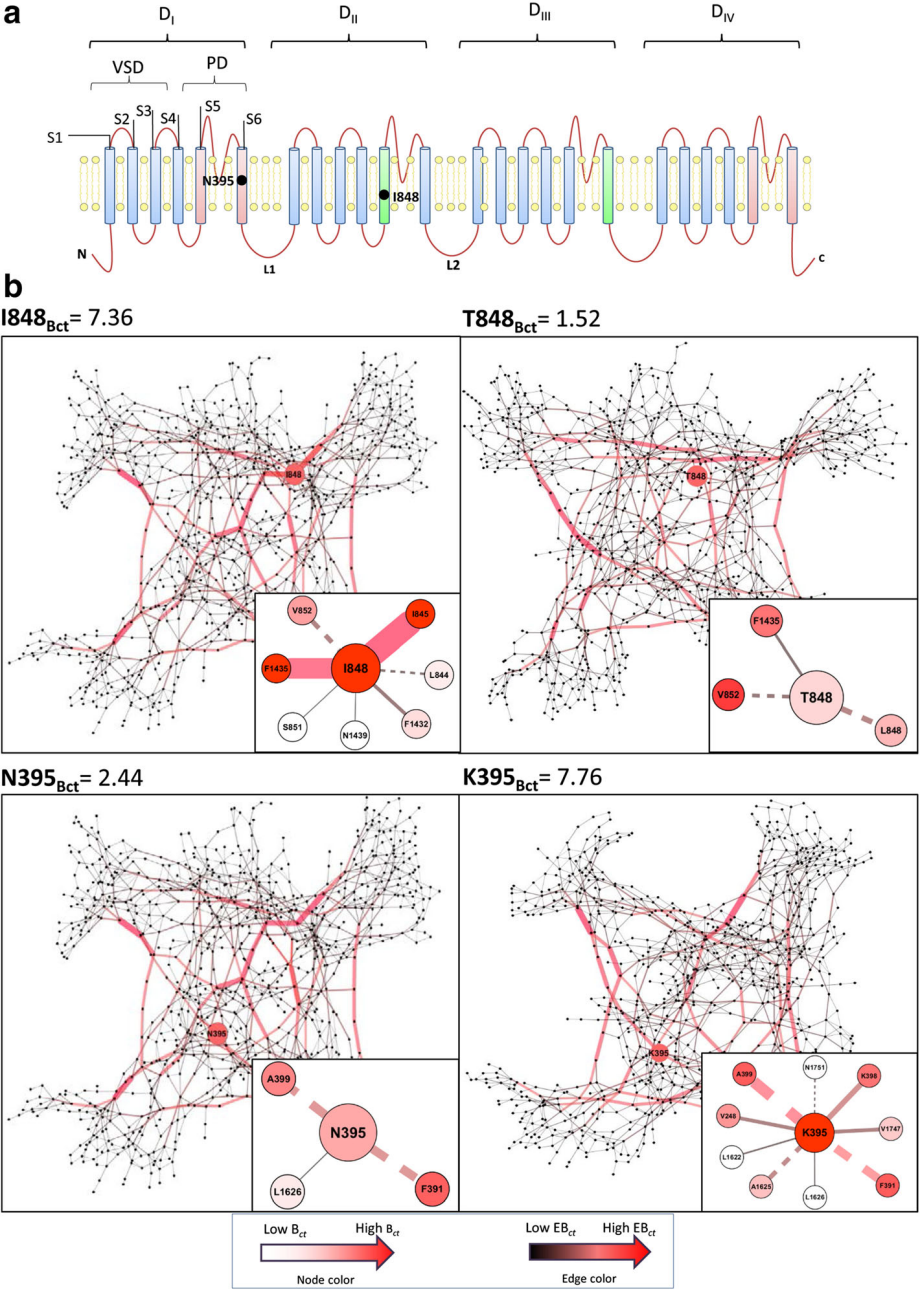
Network inter-residue connectivity of the IEM-associated mutations I848T and N395K. **a** The graph shows the NaV1.7 sodium channel topology and highlights the amino acids I848 (D_I_; S4-S5) and N395 (D_I_; S6). Inter-domain bond interaction are depicted in red for the IEM associated mutation I848T and in *green* for the IEM associated mutation N395K. **b** *Upper panels* show I848 and T848 networks, *lower panels* show N395 and K395 network. B_ct_ and EB_ct_ evidence interatomic traffic over the network. *Red-to-white* color gradient of amino acids (nodes) represents B_ct_ value (*red* represents high B_ct_ and white low B_ct_). *Red-to-black* color gradient of edges (amino acid interatomic interactions) corresponds to EB_ct_ value (*red* represents high EB_ct_ and *black* low EB_ct_). Hydrophobic bonds are showed in *solid lines* and H-bonds are indicated by *dashed lines*. I848 present high EB_*ct*_ of connecting different parts of the NaV1.7 network. *Upper right* panels show that I848 interacts through hydrophobic interactions with S4-S5 (D_II_) and pore (D_III_) through I845 and F1435 that are two residues having very high B_*ct*_ values (3.4 and 6.6, respectively) and H-bonds with V852 and L844 (I848[CO] with V852[NH]; I848[NH] with L844[CO]). Note the difference of B_ct_ of the *upper left panel* (I848B_ct_ = 7.36) compared to the *upper right panel* (T848B_ct_ = 1.52). I848T mutation interrupts the shortest paths within the network between D_II_ (S4-S5) and D_III_ (pore) and therefore ΔB_*ct*_ shifts to a negative value (-5.84). T848 interacts with F1435 through hydrophobic interactions and with S851 and L844 through H-bonds (T848[CO] with S851[NH]; T848[NH] with L844[CO]; T848[HG1] with L844[CO]). *Lower panels* show that N395 amino acid (*red*, S6 in pore module in D_I_) interacts with L1626 (S4-S5) via hydrophobic bond and via H-bonds formed by N395[CO] and A399[NH]and N395[NH]and F391[CO]. K395 mutation creates new hydrophobic bonds with V248 (S4-S5, D_I_), K398 (S6, D_I_), V1747 (S6, D_IV_), L1622 (S4-S5, D_IV_) and new H-bonds formed by K395[NZ] with N1751[CG] (S6, D_IV_) and K395[NZ] with A1625[CO] (S4-S5, D_IV_). These new bonds create a novel communication path within the network and thus increase B_ct_ value of the residue 395 (K395B_ct_ = 7.76 compared to the *left panel* N395B_ct_ = 2.44). Edge thickness are proportional to EB_*ct*_ and reveal that a high number of shortest paths pass through few edges

Figure 5 shows the network inter-residue connectivity of the IEM-associated mutations I848T and N395K, both characterized by very high ΔB_*ct*_ values. I848 is located in S4-S5 (D_II_) and I848T causes a significant hyperpolarising shift in activation, a slow deactivation and an increased response to small-ramp depolarisations in DRG nociceptors [4, 9, 17, 58, 59]. I848 is predicted to interact with S4-S5 (D_II_) and pore (D_III;_ S6) through I845 and F1435, which have with very high B_*ct*_ values (3.4 and 6.6, respectively). Upon mutation, the interatomic bond interactions between D_II_ (S4-S5) and D_III_ (pore; S6) are interrupted and therefore ΔB_*ct*_ shifts to a negative value (-5.83) showing lower EB_ct_ values (Fig. 5a and b, Additional file 16: Figure S6). Conversely, N395K mutation forms interdomain hydrophobic (S4-S5; D_I_ and D_IV_, Pore; D_IV_) and H-bonds (S4-S5; D_IV_ and S6; D_IV_), leading to a positive ΔB_*ct*_ (5) and higher EB_ct_ values.

### ΔB_ct_ distinguishes with high specificity pathogenic NaV1.7 mutations from variants not causing disease

**Fig. 6:**
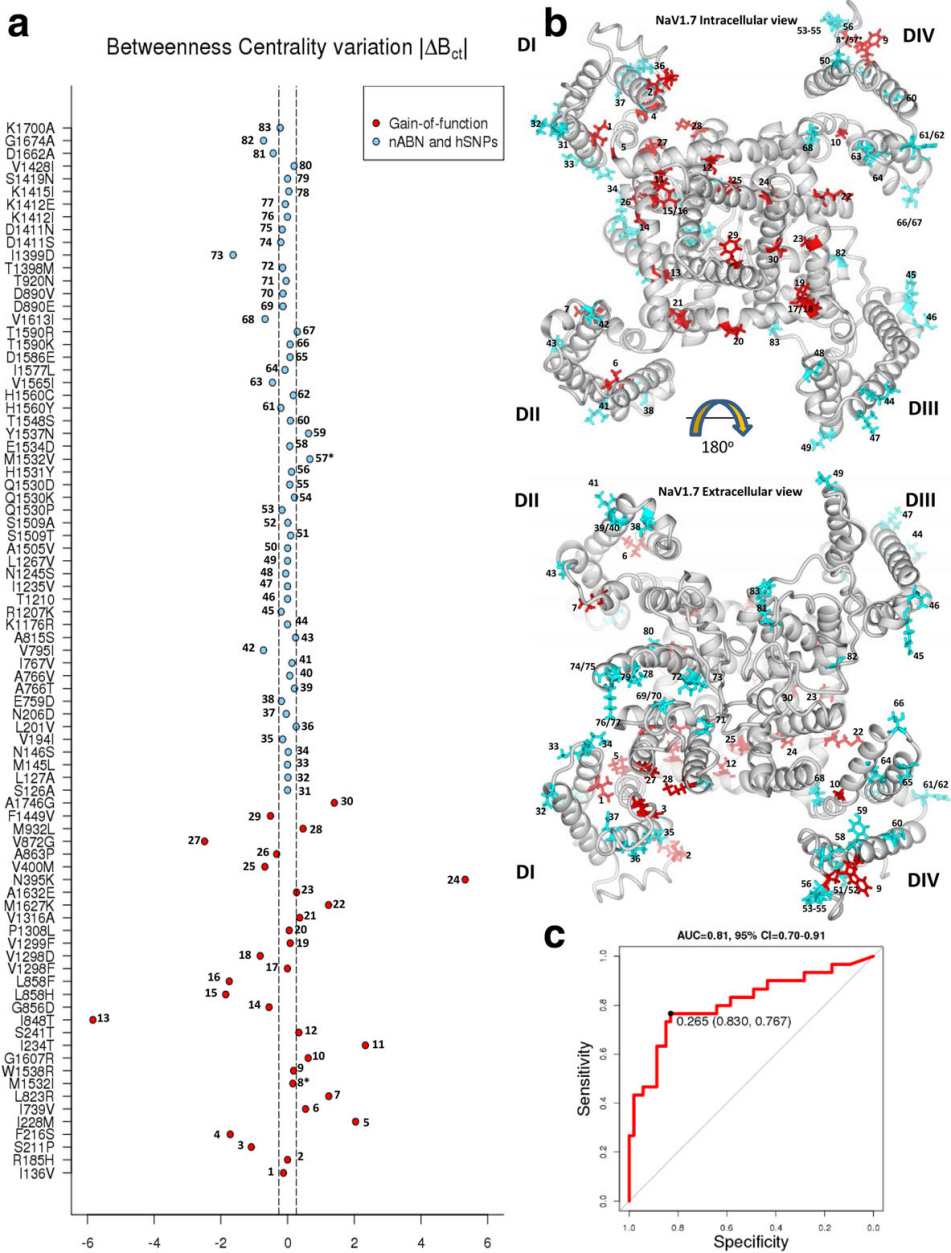
Summary of ∆B_*ct*_ values for all nABN and hSNP variants and gain-of-function mutations. **a** ∆B_*ct*_ values of all the nABN and hSNP variants (*light blue circles*) and all the gain-of-function mutations (*red circles*) analysed in this study. *Dashed lines* indicates the cut-off value (ΔB_*ct*_ ± 0.26) that maximizes sensitivity and specificity. **b** Intra- and extracellular view of the NaV1.7 and locations of amino acids affected by gain-of-function mutations (*red*) that are linked with IEM, SFN and PEPD and control group variants (nABN and hSNPs; light blue). Localization of IEM, SFN and PEPD related mutation showed in red (*red*). *The M1532 residue shares the same position with the SFN-related M1532I mutation which causes in vitro biophysics changes and M1532V variant belongs to the control group.**c **Receiver operating curve (ROC) of gain-of-function mutations and control mutations (nABN and hSNPs) as a function of ΔB_***ct***_. Using a cut-off value of ± 0.26, ΔB_***ct***_ correctly classified 44 out of 53 controls and 23 of 30 gain-of-function mutations yielding 76% sensitivity and 83% specificity. The area under the curve is 0.81 (95% Confidence Interval = 0.70 to 0.91)

The *in-silico* topological analyses described in Figures 4 and 5 was computed for all the pain disorder-related mutations (18 causing IEM, 6 causing SFN and 6 causing PEPD), and for all the 4 nABN and 49 hSNPs variants showed in Fig. 2c. The results showed that the only topological parameter that differs significantly between gain-of-function mutations and non-pathogenic amino acid changes is the |ΔB_*ct*_| value (Fig. 3). Indeed, 83% of nABN variants and hSNPs were characterized by |ΔB_*ct*_| values <0.26. The remaining 17% showed |ΔB_*ct*_| values >0.26 (*42*, V795I; *57,* M1532V; *59*, Y1537N; *63*, V1565I; *68*, V1613I; *73*, I1399D; *67*, T1590R; *81*, D1662A; *82*, G1674A) (Fig. 6a and Table 2). According to our NaV1.7 model structure, most of nABN and hSNPs, which are evolutionary variable, are located in VSD and P-loop domains and are predicted to be exposed to the lipid interface (Fig. 6b).

Twenty-three out of 30 (77%) gain-of-function NaV1.7 mutations had |ΔB_*ct*_| > 0.26 and are located in VSD, Pore and S4-S5 of D_I_, D_II_, D_III_ and D_IV_ domains (Table 1). The remaining 7 mutations (23%) had |ΔB_*ct*_| <0.26 (*1*, I136V; *2*, R185H; *8*, M1532I; *9*, W1538R; *17*, V1298F; *19*, V1299F; *20*, P1308L) (Fig. 6a and Table 1). These pathogenetic mutations with small |ΔB_*ct*_| variation are located in VSD of D_I_ (*2*, R185H) and D_III_ (*8*, M1532I; *9* W1538R) or in S4-S5 linker of D_III_ (*17*, V1298F; *19*, V1299F; *20*, P1308L), are highly evolutionary conserved residues (Additional file 5: S2 Text) and are predicted to be exposed outside the core of the channel (exception: I136V; Fig. 6b-c).

According to these results, we hypothesized that ΔB_*ct*_ might provide enough sensitivity and specificity to distinguish gain-of-function mutations from control variants. Using the cut-off value (ΔB_*ct*_ ± 0.26) that maximizes sensitivity and specificity, ΔB_*ct*_ correctly classified 44 out of 53 controls variants (nABN and hSNPs) and 23 out of 30 gain-of-function mutations, yielding 76% sensitivity and 83% specificity. The area under the ROC curve analysis for the ΔB_*ct*_ scores was 0.81 (Fig. 6c, 95% confidence interval CI = 0.70–0.91).

## Discussion

Many phenomena can be modelled as collections of elements that interact through a complex set of connections. Network theory has become one of the most successful frameworks for studying these phenomena [60] and has led to major advances in our understanding of ecological systems [61], social and communication networks [62], brain connectivity [63] and metabolic and gene regulatory pathways in living cells [64].

Using network theory, protein structure can be described as mathematical graphs [28] that represent the interatomic connections. The topological features of amino acid residues, named nodes, can be described using centrality measures that define the reciprocal relationship in terms of connectivity and capability to influence other nodes within the network. We focused on the topological analysis of NaV1.7 gain-of-function mutations identified in patients with painful disorders. We considered a homology model of the NaV1.7 pore in the closed state and calculated the interaction of the nodes within the network through several measures of topology.

Our findings show that ΔB_*ct*_ values tend to be significantly higher in NaV1.7 pain-related mutations than in control groups (nABN and hSNPs). B_*ct*_ represents the influence that the shortest communication pathways have on the overall interatomic connections. Nodes with high B_*ct*_ value could efficiently integrate signals (e.g. energy) and the reduction of B_*ct*_ value caused by single amino acid substitutions suggests that the signalling transfer capability of the network is decreased. Conversely, the increase of B_*ct*_ value suggests that a mutated node could facilitate the load transfer through the shortest communication pathways. Therefore, changes in ΔB_*ct*_ reflect increased or decreased potential for connectivity of amino acid within the protein and provides numerical values about how single amino acid substitutions might act as a bottleneck for specific nodes linking different parts of the network. Previous studies of network topological parameters revealed that effective allosteric communications can be primarily provided by structurally stable residues that exhibit high B_*ct*_ [65]. Therefore, B_*ct*_ might provide a novel and useful tool for identifying allosteric hotspots in comparison with other centrality measures as previously suggested [25, 66].

Using the cut-off value (ΔB_*ct*_ ± 0.26) that maximizes sensitivity and specificity, our data show that ΔB_*ct*_ correctly classified 44 out of 53 controls variants (nABN and hSNPs) and 23 out of 30 gain-of-function mutations, yielding 76% sensitivity and 83% specificity. The area under the ROC curve value for the ΔB_*ct*_ scores was 0.81 (Fig. 6c, 95% confidence interval CI = 0.70–0.91). By contrast, our data show that none of other topological parameters (D, CC_*ct*_, C_*ct*_, and E_*ct*_) differ significantly between controls and gain-of-function NaV1.7 mutations. Although these data suggest that the pain-related NaV1.7 gain-of-function mutations do not have significant effects on the degree of connectivity, local clustering connectivity of the neighbour nodes (i.e. their tendency to cluster together) and eccentricity (i.e. how far is each node from any other node within the network), it is important to consider that our results derive from homology modelling constructed on the closed-state pore domain of NaV1.7. A given residue may have a number of distinct interaction networks within the channel protein throughout the gating cycle, thus our modeling captures a snap shot of these interactions, and future studies are needed to further investigate interaction networks within the channel protein throughout the gating cycle.

Our NaV1.7 modeling also suggests a link between ΔB_*ct*_ value and the buried or exposed nature of an amino acid substitution. Indeed, gain-of-function mutations predicted to be buried inside or close to the core of the channel have higher |ΔB_*ct*_| than the overall mean |ΔB_*ct*_| =1.14 (*3*, S211P*; 4,* F216S; *11*, I234T; *5*, I228M*;12*, S241T; *13,* I848T; *15*, L858H; *16*, L858F; *24*, N395K; *27,* V872G; *30*, A1746G) or the cut-off value (>0.26) (*6*, I739V; *25*, V400M; *29,* F1449V). Conversely, gain-of-function mutations predicted to face the lipid interface (exception: I136V) have lower |ΔB_*ct*_| than the overall mean |ΔB_*ct*_| (1.14) (*14*, G856D; *26*, A863P; *28*, M932L; *21*, V1316A; *18*, V1298D; *10*, G1607R; *23*, A1632E; exception: M1627K) or the cut-off value |ΔB_*ct*_| (<0.26) (*17*, V1298F; *19*, V1299F; *20*, P1308L; *2*, R185H; *8*, V1532I; *9*, W1538R). Similarly, most of the control variants (nABN/hSNPs) predicted to face the lipid interface have low |ΔB_*ct*_| (<0.26) (Fig. 6b and c). Hence, in our NaV1.7 model, mutations predicted to be buried into the core of the channel show higher |ΔB_*ct*_| than those exposed at the interface of the membrane. This finding suggests that lipophilic interactions within the cell membrane may be disturbed by the mutations. Additional studies are required to more definitively assess the changes in lipophilic interactions that are produced by these mutations. Irrespective of the underlying mechanistic/molecular explanation, some pathogenic mutations would be missed by our method. Thus, ΔB_*ct*_ should be regarded as a novel *in-silico* screening tool in addition to existing common predictive algorithms (e.g. Polyphen-2 [67], SIFT [68]) that could help in selecting pathogenetic mutations for functional testing.

## Conclusions

Our findings demonstrate that most of the pathogenic NaV1.7 mutations identified in patients affected by severe painful disorders could be predicted, according to our homology modelling, to cause profound changes in the amino acid connectivity of the channel. Such modification may underpin the gain-of-function effects measurable in DRG nociceptors by electrophysiological assays. Based on these findings, we propose to consider Bct may therefore be a marker of pathogenic shift in the mutant channels, though prospective experimental studies will be required to validate its effectiveness and its biological meaning.

## Additional files

Additional file 1: S1. Text. Phylogenenetic tree of human *SCN9A* and homologous genes. (DOCX 35 kb)
Additional file 2: Table S1. NaV1.7 mutations associated to IEM, SFN and PEPD. (DOCX 50 kb)
Additional file 3: NaV1.7 pdf File. (PDB 1288 kb)
Additional file 4: Figure S1. Ramachandran plot of NaV1.7 WT. (DOCX 233 kb)
Additional file 5: S2. Text. Pairwise sequence alignment between human *SCN9A* and homologous genes. (DOCX 34 kb)
Additional file 6: Figure S2. Degree variation (∆D) in NaV1.7 mutations compared to WT. (DOCX 1691 kb)
Additional file 7: Figure S3. Clustering coefficient variation (∆CC_*ct*_) in NaV1.7 mutations compared to WT. (DOCX 2798 kb)
Additional file 8: Figure S4. Closeness Centrality variation (∆C_*ct*_) in NaV1.7 mutations compared to WT.A (DOCX 2801 kb)
Additional file 9: Figure S5. Eccentricity centrality variation (∆E_*ct*_) in NaV1.7 mutations compared to WT. (DOCX 2709 kb)
Additional file 10: S1. YASARA. Structural modelling of F216 NaV1.7 variant and their interatomic bonds. (SCE 709 kb)
Additional file 11: S2. YASARA. Structural modelling of S216 NaV1.7 variant and their interatomic bonds. (SCE 706 kb)
Additional file 12: S3. YASARA. Structural modelling of L858 NaV1.7 variant and their interatomic bonds. (SCE 707 kb)
Additional file 13: S4. YASARA. Structural modelling of H858 NaV1.7 variant and their interatomic bonds. (SCE 775 kb)
Additional file 14: S5. YASARA. Structural modelling of L1267 NaV1.7 variant and their interatomic bonds. (SCE 707 kb)
Additional file 15: S5. YASARA. Structural modelling of V1267 NaV1.7 variant and their interatomic bonds. (SCE 706 kb)
Additional file 16: Figure S6. Structural modelling variants and their interatomic bonds of I848T and N395K. (DOCX 747 kb)

## Abbreviations

B_*ct*_: Betweenness centrality
CC_*ct*_: Clustering coefficient
C_*ct*_: Closeness
D: Degree
DRG: Dorsal root ganglion
EB_*ct*_: Edge betweeness centrality
E_*ct*_: Eccentricity
H-bonds: Hydrogen bonds
hSNPs: Homologous single nucleotide polymorphisms
IEM: Inherited erythromelalgia
nABN: Variants not causing biophysical abnormalities of the channel
PD: Pore domain
PEPD: Paroxysmal extreme pain disorder
ROC: Receiver operating characteristics
SCN9A: Sodium channel, voltage-gated, type IX, alpha subunit
SFN: Painful small fibre neuropathy
VSD: Voltage-sensing domain
