# Supplementary material for: Network topology of NaV1.7 mutations in sodium channel-related painful disorders
Source: BMC Syst Biol. 2017 Feb 24;11:28. doi: 10.1186/s12918-016-0382-0 (PMC5324268; doi:10.1186/s12918-016-0382-0)

**S1 Text** Phylogenic tree between the Human *SCN9A* and other mammalian species

Phylogenic tree showing the evolutionary relationship between the Human *SCN9A* and other mammalian species. The neighbor-joining tree for the full-length proteins was generated by the ClustalW program.
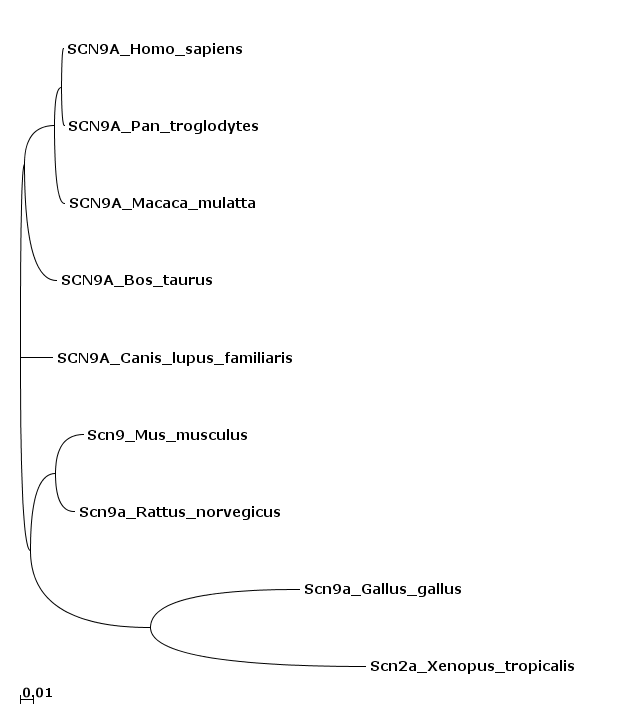

Supplement: Additional file 1: S1. — Text. Phylogenenetic tree of human SCN9A and homologous genes. (DOCX 35 kb) [file 12918_2016_382_MOESM1_ESM.docx]
