# Supplementary material for: Network topology of NaV1.7 mutations in sodium channel-related painful disorders
Source: BMC Syst Biol. 2017 Feb 24;11:28. doi: 10.1186/s12918-016-0382-0 (PMC5324268; doi:10.1186/s12918-016-0382-0)
Supplement: Additional file 2: Table S1. — NaV1.7 mutations associated to IEM, SFN and PEPD. (DOCX 50 kb) [file 12918_2016_382_MOESM2_ESM.docx]

| **Disease** | **Mutation** | **Reference** |
| --- | --- | --- |
| IEM | I136V  S211P  F216S  L823R  W1538R | (Cheng *et al.*, 2008; Lee *et al.*, 2007; Wu *et al.*, 2013)  (Estacion *et al.*, 2010)  (Drenth *et al.*, 2005; Choi *et al.*, 2006)  (Lampert *et al.*, 2009; Takahashi *et al.*, 2007)  (Cregg *et al.*, 2013) |
|  | I234T  S241T  I848T  L858H  L858F  A863P  P1308L  V1316A | (Ahn *et al.*, 2010)  (Lampert *et al.*, 2006; Michiels *et al.*, 2005; Yang *et al.*, 2012)  (Cummins et al. 2004; Han et al. 2009; Theile et al. 2011;  Wu et al. 2013; Yang et al. 2004)  (Cummins *et al.*, 2004; Theile *et al.*, 2011; Yang *et al.*, 2004)  (Cheng *et al.*, 2011; Drenth *et al.*, 2005; Han *et al.*, 2006)  (Harty *et al.*, 2006)  (Cheng *et al.*, 2010)  (Estacion *et al.*, 2013; Wu *et al.*, 2013) |
|  | N395K  V400M  V872G  F1449V  A1746G | (Sheets *et al.*, 2007; Drenth *et al.*, 2005)  (Fischer *et al.*, 2009; Yang *et al.*, 2012)  (Choi *et al.*, 2009)  (Yang *et al.*, 2012; Dib-Hajj *et al.*, 2005)  (Cregg *et al.*, 2013) |
| SFN | R185H  I228M  I739V  G856DM1532I | (Faber *et al.*, 2012)  (Faber et al. 2012; Estacion et al. 2011;)  (Faber *et al.*, 2012; Han *et al.*, 2012)  (Hoeijmakers *et al.*, 2012b)  (Faber *et al.*, 2012) |
|  | M932L | (Faber *et al.*, 2012) |
| PEPD | V1298D  V1298F  V1299F  G1607R  M1627K  A1632E | (Fertleman *et al.*, 2006)  (Fertleman *et al.*, 2006; Jarecki *et al.*, 2008; Cheng *et al.*, 2010)  (Fertleman *et al.*, 2006; Jarecki *et al.*, 2008; Theile *et al.*, 2011)  (Choi *et al.*, 2011)  (Theile and Cummins, 2011; Fertleman *et al.*, 2006; Dib-Hajj *et al.*, 2008; Theile *et al.*, 2011)  (Estacion *et al.*, 2008) |

**Table S1** NaV1.7 mutations associated to IEM, SFN and PEPD
