# Supplementary material for: Network topology of NaV1.7 mutations in sodium channel-related painful disorders
Source: BMC Syst Biol. 2017 Feb 24;11:28. doi: 10.1186/s12918-016-0382-0 (PMC5324268; doi:10.1186/s12918-016-0382-0)

**S1 Figure**

Ramachandran plot illustrated the backbone confirmation for the modeled NaV1.7. Ramachandran plot for the NaV1.7 model showed 88.5% (882 residues) in most favored region, 9% (90 residues) in allowed region and 2.5% (25 regions) in outlier region indicate the quality of the structure predicted by the RAMPAGE server.


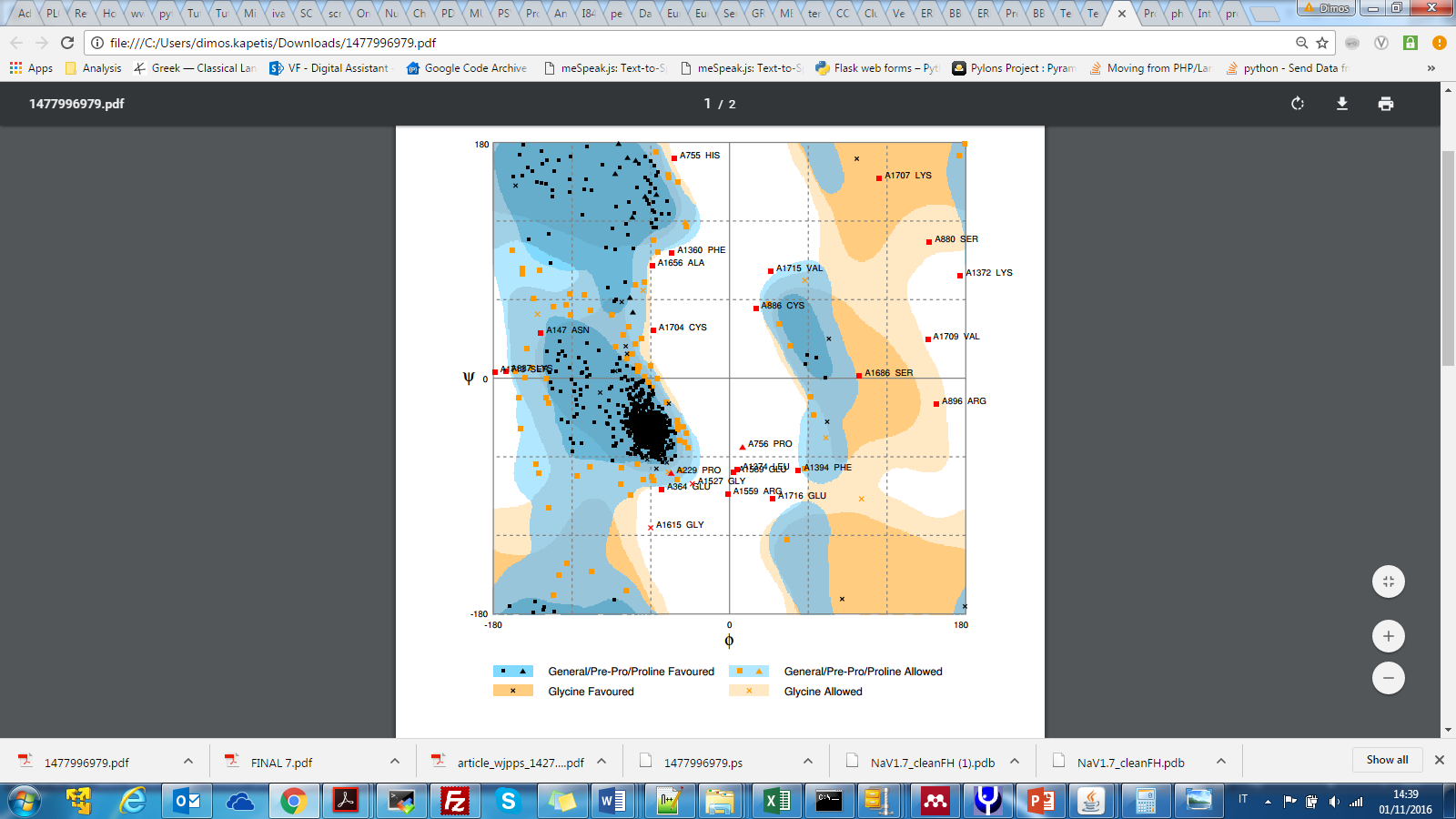

Supplement: Additional file 4: Figure S1. — Ramachandran plot of NaV1.7 WT. (DOCX 233 kb) [file 12918_2016_382_MOESM4_ESM.docx]
