# Supplementary material for: Network topology of NaV1.7 mutations in sodium channel-related painful disorders
Source: BMC Syst Biol. 2017 Feb 24;11:28. doi: 10.1186/s12918-016-0382-0 (PMC5324268; doi:10.1186/s12918-016-0382-0)
Supplement: Additional file 5: S2. — Text. Pairwise sequence alignment between human SCN9A and homologous genes. (DOCX 34 kb) [file 12918_2016_382_MOESM5_ESM.docx]

**S2 Text** Human *SCN9A* pairwise sequence alignment and mammalian species

*SCN9A* pairwise sequence alignment between *Homo sapiens* and mammalian species showing >90% of nucleotide sequence identity (*Pan troglodytes*, Pt; *Macacca Mulata*, Mmu; *Canis familiaris*, Cf; *Bos taurus*, Bt) whereas *Mus Musculus* (Mm), *Rattus Norvegicus* (Rn), *Gallus gallus* (Gn) and *Xenopus tropicalis* (Xn) showing < 90% sequence identity. Residues located in the four transmembrane domains (D_I_-D_IV_), highlighted in red (associated with IEM, SFN and PEPD) and light blue (nABN and hSNPs), were selected to perform *in-silico* mutagenesis.

**Consensus (cons) alignment** BAD AVG GOOD

SCN9A-Hs       1 MA--M--LPPPGPQSFVHFTKQSLALIEQRIAERKSKEPKEE   38 
SCN9A-Pt       1 MA--M--LPPPGPQSFVHFTKQSLALIEQRIAERKSKEPKEE   38 
SCN9A-Mmu      1 MA--M--LPPPGPQSFVHFTKQSLALIEQRIAERKSKEPKEE   38 
SCN9A-Cf       1 MA--T--LPPPGPQSFVYFTKQSLALIEQRIAEGKTKEPKEE   38 
SCN9A-Bt       1 MA--M--LPPPGPQSFVFFTKQSLALIEQRIAEGKAKEPKEE   38 
Scn9-Mm        1 MA--M--LPPPGPQSFVHFTKQSLALIEQRISEEKAKGHKDE   38 
Scn9a-Rn       1 MA--M--LPPPGPQSFVHFTKQSLALIEQRISEEKAKEHKDE   38 
Scn9a-Gg       1 MAQAM--LVPPGSDCFFYFTRESLAAIEKRIADEKSNRDKDE   40 
Scn2a-Xt       1 ME--QLRIVPPGPDSFRYFTRESLKAIEKRISEEKENNAKQE   40 

cons           1 *      : ***.:.* .**::**  **:**:: * :  *:*   42 


SCN9A-Hs      39 KKDDD---EEAP-----KPSSDLEAGKQLPFIYGDIPPGMVS   72 
SCN9A-Pt      39 KKDDD---EEAPKPSKPKPSSDLEAGKQLPFIYGDIPPGMVS   77 
SCN9A-Mmu     39 KKDDD---EEAP-----KPSSDLEAGKQLPFIYGDIPPGMVS   72 
SCN9A-Cf      39 KKDDD---EEGP-----RPSSDLEAGKQLPFIYGDIPPGMVS   72 
SCN9A-Bt      39 KKDDD---DEGP-----KPSSDLEAGKQLPFIYGDIPPGMVS   72 
Scn9-Mm       39 KKDDE---EEGP-----KPSSDLEAGKQLPFIYGDIPPGMVS   72 
Scn9a-Rn      39 KKDDE---EEGP-----KPSSDLEAGKQLPFIYGDIPPGMVS   72 
Scn9a-Gg      41 HKDDGGHEEVQP-----TPNNDLEAGKTLPFIYGDIPPEMVS   77 
Scn2a-Xt      41 QKEGE---ENGP-----KPNRDLEAGQSLPFIYGDIPPRMVS   74 

cons          43 :*:.    :  *      *. *****: ********** ***   84 


SCN9A-Hs      73 EPLEDLDPYYADKKTFIVLNKGKTIFRFNATPALYMLSPFSP  114 
SCN9A-Pt      78 EPLEDLDPYYADKKTFIVLNKGKTIFRFNATPALYMLSPFSP  119 
SCN9A-Mmu     73 EPLEDLDPYYADKKTFIVLNKGKTIFRFNATPALYMLSPFSP  114 
SCN9A-Cf      73 EPLEDLDPYYADKKTFIVLNKGKAIFRFNATPALYMLSPFSP  114 
SCN9A-Bt      73 EPLEDLDPYYADKKTFIVLNKGKAIFRFNATPALYMLSPFSP  114 
Scn9-Mm       73 EPLEDLDPYYADKKTFIVLNKGKAIFRFNATPALYMLSPFSP  114 
Scn9a-Rn      73 EPLEDLDPYYADKKTFIVLNKGKAIFRFNATPALYMLSPFSP  114 
Scn9a-Gg      78 EPLEDLDPYYINKKTFIVLNKGKTIFRFSATSALYLLSPFNV  119 
Scn2a-Xt      75 EPLEDLDPYYANQKTFIVLNKGKTLFRFSATSALYILTPFNP  116 

cons          85 ********** ::**********::***.**.***:*:**.   126

**126-127 136 145-146**
SCN9A-Hs     115 LRRISIKILVHSLFSMLIMCTILTNCIFMTMNNPPDWTKNVE  156 
SCN9A-Pt     120 LRRISIKILVHSLFSMLIMCTILTNCIFMTMNNPPDWTKNVE  161 
SCN9A-Mmu    115 LRRISIKILVHSLFSMLIMCTILTNCIFMTMSNPPDWTKNVE  156 
SCN9A-Cf     115 LRRISIKILVHSLFSMLIMCTILTNCIFMTMSNPPDWTKNVE  156 
SCN9A-Bt     115 LRRISIKILVHAAFSMLIMCTILTNCIFMTMSNPPDWTKNVE  156 
Scn9-Mm      115 LRRISIKILVHSLFSMLIMCTILTNCIFMTMSNPPDWTKNVE  156 
Scn9a-Rn     115 LRRISIKILVHSLFSMLIMCTILTNCIFMTLSNPPEWTKNVE  156 
Scn9a-Gg     120 IRRVAIKILVHSLFSMLIMLTILTNCVFMTWSKLPEWTKNVE  161 
Scn2a-Xt     117 VRQAAIKILVHSLFNMLIMCTILTNCVFMTLSNPPEWTKNVE  158 

cons         127 :*: :******: *.**** ******:*** .: *:******  168 

 **185 194**
SCN9A-Hs     157 YTFTGIYTFESLVKILARGFCVGEFTFLRDPWNWLDFVVIVF  198 
SCN9A-Pt     162 YTFTGIYTFESLVKILARGFCVGEFTFLRDPWNWLDFVVIVF  203 
SCN9A-Mmu    157 YTFTGIYTFESLVKILARGFCVGEFTFLRDPWNWLDFIVIVF  198 
SCN9A-Cf     157 YTFTGIYTFESLVKILARGFCVGEFTFLRDPWNWLDFVVIVF  198 
SCN9A-Bt     157 YTFTGIYTFESLVKILARGFCVGEFTFLRDPWNWLDFIVIVF  198 
Scn9-Mm      157 YTFTGIYTFESLIKILARGFCVGEFTFLRDPWNWLDFVVIVF  198 
Scn9a-Rn     157 YTFTGIYTFESLIKILARGFCVGEFTFLRDPWNWLDFVVIVF  198 
Scn9a-Gg     162 YTFTGIYTFEFLVKILARGFCIDDFTCLRDPWNWLDFVVISF  203 
Scn2a-Xt     159 YTFTGIYTLESLIKILARGFCLEKFTFLRDPWNWLDFSVIVL  200 

cons         169 ********:* *:********: .** ********** ** :  210 

 **201** **206 211 216 228 234**
SCN9A-Hs     199 AYLTEFVNLGNVSALRTFRVLRALKTISVIPGLKTIVGALIQ  240 
SCN9A-Pt     204 AYVTEFVDLGNVSALRTFRVLRALKTISVIPGLKTIVGALIQ  245 
SCN9A-Mmu    199 AYLTEFVNLGNVSALRTFRVLRALKTISVIPGLKTIVGALIQ  240 
SCN9A-Cf     199 AYVTEFVDLGNVSALRTFRVLRALKTISVIPGLKTIVGALIQ  240 
SCN9A-Bt     199 AYLTEFVNLGNVSALRTFRVLRALKTISVIPGLKTIVGALIQ  240 
Scn9-Mm      199 AYLTEFVNLGNVSALRTFRVLRALKTISVIPGLKTIVGALIQ  240 
Scn9a-Rn     199 AYLTEFVNLGNVSALRTFRVLRALKTISVIPGLKTIVGALIQ  240 
Scn9a-Gg     204 AYVTEFVDLGNVSALRTFRVLRALKTISVIPGLKTIVGALIQ  245 
Scn2a-Xt     201 AYVTEFVNLGNVSALRTFRVLRALKTISVIPGLKTIVGALIQ  242 

cons         211 **:****:**********************************  252 

 **241**
SCN9A-Hs     241 SVKKLSDVMILTVFCLSVFALIGLQLFMGNLKHKCFRNSLEN  282 
SCN9A-Pt     246 SVKKLSDVMILTVFCLSVFALIGLQLFMGNLKHKCFRNSLEN  287 
SCN9A-Mmu    241 SVKKLSDVMILTVFCLSVFALIGLQLFMGNLKHKCVQNSLVN  282 
SCN9A-Cf     241 SVKKLSDVMILTVFCLSVFALIGLQLFMGNLKHKCLRDPLDD  282 
SCN9A-Bt     241 SVKKLSDVMILTVFCLSVFALIGLQLFMGNLKHKCVQSSLAN  282 
Scn9-Mm      241 SVKKLSDVMILTVFCLSVFALIGLQLFMGNLKHKCFRKDLEQ  282 
Scn9a-Rn     241 SVKKLSDVMILTVFCLSVFALIGLQLFMGNLKHKCFRKELEE  282 
Scn9a-Gg     246 SVKKLSDVMILTVFCLSVFALIGLQLFMGNLKNKCLLWPSRN  287 
Scn2a-Xt     243 SVKKLSDVMILTVFCLSVFALIGLQLFMGHLRNKCLLWPYNS  284 

cons         253 *****************************:*::**.     .  294 


SCN9A-Hs     283 NETLESIMNTLESEE--------D--F-------------RK  301 
SCN9A-Pt     288 NETLESIMNTLESEE--------D--FRIDREWETWYLKKSE  319 
SCN9A-Mmu    283 NETLESIMNTLESEE--------D--F-------------RK  301 
SCN9A-Cf     283 NETLTSLLDTLEEED---YKSKNQ--H-------------SK  306 
SCN9A-Bt     283 NETMENILNTLDEEE-----------Y-------------AK  300 
Scn9-Mm      283 NETLENGHKGYQRKC--------KVKT-------------MG  303 
Scn9a-Rn     283 NETLESIMNTAESEE--------E--L-------------KK  301 
Scn9a-Gg     288 STSFEKYLAPYFNDTVFDWAAYIE--N-------------ES  314 
Scn2a-Xt     285 SVDISL------YEH--------F--N-------------ES  297 

cons         295 .  :         .                              336 


SCN9A-Hs     302 YFYYLEGSKDALLCGFSTDSGQCPEGYTCVKIGRNPDYGYTS  343 
SCN9A-Pt     320 YFYYLEGSKDALLCGFSTDSGQCPEGYTCVKIGRNPDYGYTS  361 
SCN9A-Mmu    302 YFYYLEGSKDALLCGFSTDSGQCPEGYTCMKIGRNPDYGYTS  343 
SCN9A-Cf     307 YFYYLEGSKDALLCGLSTDSGQCPEGYKCVKAGRNPDYGYTS  348 
SCN9A-Bt     301 YFYYLEGSKDALLCGFSSDSGQCPEGYTCKKIGRNPDYGYTS  342 
Scn9-Mm      304 YFYYLEGSKDALLCGFSTDSGQCPEGYECVTAGRNPDYGYTS  345 
Scn9a-Rn     302 YFYYLEGSKDALLCGFSTDSGQCPEGYICVKAGRNPDYGYTS  343 
Scn9a-Gg     315 HFYRLEGQKDYLLCGNSSDAGKCPEEFICVKAGRNPNYGYTS  356 
Scn2a-Xt     298 LFYKLENQKDPLVCGNSSDTGKCPEGYVCVKAGRNPNYGYTS  339 

cons         337  ** **..** *:** *:*:*:*** : * . ****:*****  378 


SCN9A-Hs     344 FDTFSWAFLALFRLMTQDYWENLYQQTLRAAGKTYMIFFVVV  385 
SCN9A-Pt     362 FDTFSWAFLALFRLMTQDYWENLYQQTLRAAGKTYMIFFVVV  403 
SCN9A-Mmu    344 FDTFSWAFLALFRLMTQDYWENLYQQTLRAAGKTYMIFFVVV  385 
SCN9A-Cf     349 FDTFSWAFLALFRLMTQDYWENLYQQTLRAAGKTYMIFFVVV  390 
SCN9A-Bt     343 FDTFSWAFLALFRLMTQDYWENLYQQTLRAAGKTYMIFFVVV  384 
Scn9-Mm      346 FDTFGWAFLALFRLMTQDYWENLYQQTLRAAGKTYMIFFVVV  387 
Scn9a-Rn     344 FDTFSWAFLALFRLMTQDYWENLYQQTLRAAGKTYMIFFVVV  385 
Scn9a-Gg     357 FDTFSWAFLSLFRLMTQDYWENLYQLTLRAVGKTYMIFFVLV  398 
Scn2a-Xt     340 FDTFNWAFLSLFRLMTQDCWENLYQLTLRAAGKTYMIFFVLV  381 

cons         379 ****.****:******** ****** ****.*********:*  420 

 **395 400**
SCN9A-Hs     386 IFLGSFYLINLILAVVAMAYEEQNQANIEEAKQKELEFQQML  427 
SCN9A-Pt     404 IFLGSFYLINLILAVVAMAYEEQNQANIEEAKQKELEFQQML  445 
SCN9A-Mmu    386 IFLGSFYLINLILAVVAMAYEEQNQANIEEAKQKELEFQQML  427 
SCN9A-Cf     391 IFLGSFYLINLILAVVAMAYEEQNQANIEEARQKELEFQQML  432 
SCN9A-Bt     385 IFLGSFYLINLILAVVAMAYEEQNQANIEEARQKELEFQQML  426 
Scn9-Mm      388 IFLGSFYLINLILAVVAMAYEEQNQANIEEAKQKELEFQQML  429 
Scn9a-Rn     386 IFLGSFYLINLILAVVAMAYEEQNQANIEEAKQKELEFQQML  427 
Scn9a-Gg     399 IFLGSFYLINLILAVVAMAYEEQNQATMVEAEQREADLQQML  440 
Scn2a-Xt     382 IFLGSFYLVNLILAVVAMAYEEQNQATIEEAVQKEAEFQ---  420 

cons         421 ********:*****************.: ** *:* ::*     462 


SCN9A-Hs     428 DRLKKEQEEAEAIAAAAAEYT--SIRRSRIMG---LSESSSE  464 
SCN9A-Pt     446 DRLKKEQEEAEAIAAAAAEYT--SIRRSRIMG---LSESSSE  482 
SCN9A-Mmu    428 DRLKKEQEEAEAIAAAAAEYT--SIRRSRIMG---LSESSSE  464 
SCN9A-Cf     433 DRLKKEQEEAEAIAIAAAEYT--SIGRSRMMG---FSESSSE  469 
SCN9A-Bt     427 DRLKKEQEEAEAIALAAAEYT--SIGRSRIMG---LSESSSE  463 
Scn9-Mm      430 DRLKKEQEEAEVVFIITNTKA--KQVGQRIMG---LSESSSE  466 
Scn9a-Rn     428 DRLKKEQEEAEAIAAAAAEFT--SIGRSRIMG---LSESSSE  464 
Scn9a-Gg     441 EQLKKQQEEAQAIAAAAVEMT--EFGGE--SG---PSDSSSE  475 
Scn2a-Xt     421 -RLKKQQEEAQALAAAVADVFGESQELSEEVGLEDASECSST  461 

cons         463  :***:****:.:   .      .   .   *    *:.**   504 


SCN9A-Hs     465 TSKLSSKSAKERRNRRKKKNQKKLSSGEEKGDAEKLSKSESE  506 
SCN9A-Pt     483 TSKLSSKSAKERRNRRKKKNQKKLSSGEEKGDAEKLSKSESE  524 
SCN9A-Mmu    465 TSKLSSKSAKERRNRRKKKNQKKLSSGEEKGDAEKLSKSDSE  506 
SCN9A-Cf     470 TSKLSSKSAKERRNRRKKRNQKKLSSGEEKGDNEKLSKSESE  511 
SCN9A-Bt     464 TSKLSSKSAKERRNRRRKKNQKKLSSGEEKGDDEKLSKSESE  505 
Scn9-Mm      467 TSRLSSKSAKERRNRRKKKKQK-LSSGEEKGDDEKLSKSGSE  507 
Scn9a-Rn     465 TSRLSSKSAKERRNRRKKKKQK-MSSGEEKGDDEKLSKSGSE  505 
Scn9a-Gg     476 ASKFSSKSAKERRNRRKKRRQREHSGEEDNMKDTKLSKSESD  517 
Scn2a-Xt     462 VSKLSSKSAKERRNWRKQRKQVAHCDGQEKGDKEKCYKSGSE  503 

cons         505 .*::********** *:::.*   .. ::: .  *  ** *:  546 


SCN9A-Hs     507 DSIRRKSFHLGVEGHRRAHEKRLSTPNQSPLSIRGSLFSARR  548 
SCN9A-Pt     525 DSIRRKSFHLGVEGHRRAHEKRLSTPNQSPLSIRGSLFSARR  566 
SCN9A-Mmu    507 ENIRRKSFHLGVEGHRRAHEKRLSTPSQSPLSIRGSLFSARR  548 
SCN9A-Cf     512 ESIRRQSFHLGVEGHRRAREKRLSTPNQSPLSIRGSLFSGRR  553 
SCN9A-Bt     506 ESIRRKSFHLGVEGHRRAREKRLSTPNQSPLSIRGSLFSARR  547 
Scn9-Mm      508 ESIRKKSFHLGVEGHHRAREKRLSTPNQSPLSIRGSLFSARR  549 
Scn9a-Rn     506 ESIRKKSFHLGVEGHHRTREKRLSTPNQSPLSIRGSLFSARR  547 
Scn9a-Gg     518 GSIRRKGFRFSFDGNKLAYGTRLTSPHQSLLSIRGSLFSPRR  559 
Scn2a-Xt     504 DSIKRKGLNFTREGRRLEEYRKSSMPHQSLLSIQGPVFSPRR  545 

cons         547  .*:::.:.:  :*.:     : : * ** ***:*.:** **  588 


SCN9A-Hs     549 SSRTSLFSFKGRGRDIGSETEFADDEHSIFGDNESRRGSLFV  590 
SCN9A-Pt     567 SSRTSLFSFKGRGRDIGSETEFADDEHSIFGDNESRRGSLFV  608 
SCN9A-Mmu    549 SSRTSLFSFKGRGRDIGSETEFADDEHSIFGDNESRRGSLFV  590 
SCN9A-Cf     554 SSRTSLFSFKGRGKDIGSETEFADDEHSIFGDSESRRGSLFV  595 
SCN9A-Bt     548 SSRTSLFSFKGRGRDIGSETEFADDEHSIFGDNESRRGSLFV  589 
Scn9-Mm      550 SSRTSLFSFKGRGRDLGSETEFADDEHSIFGDNESRRGSLFV  591 
Scn9a-Rn     548 SSRTSLFSFKGRGRDLGSETEFADDEHSIFGDNESRRGSLFV  589 
Scn9a-Gg     560 SSRTSLFSFRDHGKEIGSENDFADDEHSTFDDNGSRRGSLFV  601 
Scn2a-Xt     546 NSKTSIFSFKDRAKDVGSENDFADDERSTFE-EDSRRDPLFV  586 

cons         589 .*:**:***:.:.:::***.:*****:* *  . ***..***  630 


SCN9A-Hs     591 PHRPQERRSSNISQASRS---PP---MLPVNGKMHSAVDCNG  626 
SCN9A-Pt     609 PHRPQERRSSNISQASRS---PP---MLPVNGKMHSAVDCNG  644 
SCN9A-Mmu    591 PHRPQERRSSNISQASRS---PP---ILPVNGKMHSAVDCNG  626 
SCN9A-Cf     596 PHRPRERRSSNISQASRS---PP---VLPVNGKMHSAVDCNG  631 
SCN9A-Bt     590 PHRPRERRSSNISQASRS---PP---VLPVNGKMHSAVDCNG  625 
Scn9-Mm      592 PHRPRERRSSNISQASRS---PP---VLPVNGKMHSAVDCNG  627 
Scn9a-Rn     590 PHRPRERRSSNISQASRS---PP---VLPVNGKMHSAVDCNG  625 
Scn9a-Gg     602 PLRHSERRGSNISQASRPSRRLT---LFPVNGKMHSTVDCNG  640 
Scn2a-Xt     587 PGRHRARRNSNISQTSRS---SRILPLLSMNGKMHSTVDCNG  625 

cons         631 * *   **.*****:**.        ::.:******:*****  672 


SCN9A-Hs     627 VVSLVDGRSALMLPNGQLLPE-----------GTTNQIH-KK  656 
SCN9A-Pt     645 VVSLVDGRSALMLPNGQLLPEVIIDKATSDDSGTTNQIH-KK  685 
SCN9A-Mmu    627 VVSLVDGRSALMLPNGQLLPE-----------GTTNQIH-KK  656 
SCN9A-Cf     632 VVSLVDGPSALMLPNGQLLPEVIIDKATSDDSGTTNQIH-KK  672 
SCN9A-Bt     626 VVSLVDGPSALMLPNGQLLPEVIIDKATSDDSGTTNQIH-KK  666 
Scn9-Mm      628 VVSLVDGPSALMLPNGQLLPE-----------GTTNQMR-KK  657 
Scn9a-Rn     626 VVSLVDGPSALMLPNGQLLPEVIIDKATSDDSGTTNQMR-KK  666 
Scn9a-Gg     641 VVSLVDRPPCLLSPTGQLLPELIIDKPTTDDNSTTTEMEIKK  682 
Scn2a-Xt     626 VVSL----------------------------GTTTETD-RK  638 

cons         673 ****                            .**.:   :*  714 


SCN9A-Hs     657 RRCSSYLLSEDMLNDPNLRQRAMSRASILTNTVEELEESRQK  698 
SCN9A-Pt     686 RRCSSYLLSEDMLNDPNLRQRAMSRASILTNTVEELEESRQK  727 
SCN9A-Mmu    657 RRCSSYLLSEDMLNDPNLRQRAMSRASILTNTVEELEESRQK  698 
SCN9A-Cf     673 RRSSSYLLSEDMLNDPNLRQRAMSRVSILTNTVEELEESRQK  714 
SCN9A-Bt     667 RRHSSYLLSEDMLNDPNLRQRAMSRASILTNTVEELEESRQK  708 
Scn9-Mm      658 RLSSSYFLSEDMLNDPHLRQRAMSRASILTNTVEELEESRQK  699 
Scn9a-Rn     667 RLSSSYFLSEDMLNDPHLRQRAMSRASILTNTVEELEESRQK  708 
Scn9a-Gg     683 RRSSSYQIPMDLLEDPNLRQRAMSIAGIITNTMEELEESRQK  724 
Scn2a-Xt     639 RRPSSYKISMDLMEDPSLRQRAQSIASILTNTMEELQESRRK  680 

cons         715 *  *** :. *:::** ***** * ..*:***:***:***:*  756 

**739**
SCN9A-Hs     699 CPPWWYRFAHKFLIWNCSPYWIKFKKCIYFIVMDPFVDLAIT  740 
SCN9A-Pt     728 CPPWWYRFAHKFLIWNCSPYWIKFKKCIYFIVMDPFVDLAIT  769 
SCN9A-Mmu    699 CPPWWYRFAHKFLIWNCSPYWIKFKKCIYFIVMDPFVDLAIT  740 
SCN9A-Cf     715 CPPWWYRFAHTFLIWNCSPYWIKFKKLVYFIVMDPFVDLAIT  756 
SCN9A-Bt     709 CPPWWYRFAHTFLIWNCSPYWIKFKKFIYFIVMDPFVDLAIT  750 
Scn9-Mm      700 CPPWWYRFAHTFLIWNCSPYWIKFKKFIYFIVMDPFVDLAIT  741 
Scn9a-Rn     709 CPPWWYRFAHTFLIWNCSPYWIKFKKLIYFIVMDPFVDLAIT  750 
Scn9a-Gg     725 CPPCWYKFAHTYLIWNCCEVWLKVKSVVSFIVMDPLVDLAIT  766 
Scn2a-Xt     681 CPSCWYKFANCFLIWDCCEAWLKVKRIVKLIVMDPFVDLAIT  722 

cons         757 **. **:**: :***:*.  *:*.*  : :*****:******  798 

 **759 766-7**
SCN9A-Hs     741 ICIVLNTLFMAMEHHPMTEEFKNVLAIGNLVFTGIFAAEMVL  782 
SCN9A-Pt     770 ICIVLNTLFMAMEHHPMTEEFKNVLAIGNLVFTGIFAAEMVL  811 
SCN9A-Mmu    741 ICIVLNTLFMAMEHHPMTEEFKNVLAIGNLVFTGIFAAEMVL  782 
SCN9A-Cf     757 ICIVLNTLFMAMEHHPMTDEFKNVLTVGNLVFTGIFAAEMVL  798 
SCN9A-Bt     751 ICIVLNTLFMAMEHHPMTEEFKNVLVVGNLVFTGIFAAEMVL  792 
Scn9-Mm      742 ICIVLNTLFMAMEHHPMTDEFKNVLAVGNLVFTGIFAAEMVL  783 
Scn9a-Rn     751 ICIVLNTLFMAMEHHPMTEEFKNVLAVGNLIFTGIFAAEMVL  792 
Scn9a-Gg     767 ICIILNTLFMAMEHYPMTETFNNTLKVGNQVFTGIFAAEMVL  808 
Scn2a-Xt     723 ISIVLNTIFMAVEHAHMTPYFISVLTTGNQVFTGIFTAEMVL  764 

cons         799 *.*:***:***:**  **  * ..*  ** :*****:*****  840 

 **795 815 823**
SCN9A-Hs     783 KLIAMDPYEYFQVGWNIFDSLIVTLSLVELFLADVEGLSVLR  824 
SCN9A-Pt     812 KLIAMDPYEYFQVGWNIFDSLIVTLSLVELFLADVEGLSVLR  853 
SCN9A-Mmu    783 KLIAMDPYEYFQVGWNIFDSLIVTLSLVELFLADVEGLSVLR  824 
SCN9A-Cf     799 KLIAMDPYEYFQVGWNIFDSLIVTLSLVELFLADVEGLSVLR  840 
SCN9A-Bt     793 KLIAMDPYEYFQIGWNIFDSLIVTLSLVELFLSDVEGLSVLR  834 
Scn9-Mm      784 KLIAMDPYEYFQVGWNIFDSLIVTLSLVELFLADVEGLSVLR  825 
Scn9a-Rn     793 KLIAMDPYEYFQVGWNIFDSLIVTLSLIELFLADVEGLSVLR  834 
Scn9a-Gg     809 KIIAMDPFYYFQVGWNIFDSFIVTLSLVELFLANVDGLSVLR  850 
Scn2a-Xt     765 KLIALDPYYYFQEGWNIFDGLIVSLSLMELGLSSTGGFSVLR  806 

cons         841 *:**:**: *** ******.:**:***:** *:.. *:****  882 

 **848 856 858 863**
SCN9A-Hs     825 SFRLLRVFKLAKSWPTLNMLIKIIGNSVGALGNLTLVLAIIV  866 
SCN9A-Pt     854 SFRLLRVFKLAKSWPTLNMLIKIIGNSVGALGNLTLVLAIIV  895 
SCN9A-Mmu    825 SFRLLRVFKLAKSWPTLNMLIKIIGNSVGALGNLTLVLAIIV  866 
SCN9A-Cf     841 SFRLLRVFKLAKSWPTLNMLIKIIGNSVGALGNLTLVLAIIV  882 
SCN9A-Bt     835 SFRLLRVFKLAKSWPTLNMLIKIIGNSVGALGNLTLVLAIIV  876 
Scn9-Mm      826 SFRLLRVFKLAKSWPTLNMLIKIIGNSVGALGNLTLVLAIIV  867 
Scn9a-Rn     835 SFRLLRVFKLAKSWPTLNMLIKIIGNSVGALGNLTLVLAIIV  876 
Scn9a-Gg     851 SFRLLRVFKLAKSWPTLNMLIKIIGNSVGALGNLTLVLAIIV  892 
Scn2a-Xt     807 SFRLLRVFKLAKSWPTLNKLIKIIGNSVGALGNLTLVLAIIV  848 

cons         883 ****************** ***********************  924 

 **872 890**
SCN9A-Hs     867 FIFAVVGMQLFGKSYKECVCKINDDCTLPRWHMNDFFHSFLI  908 
SCN9A-Pt     896 FIFAVVGMQLFGKSYKECVCKINDDCTLPRWHMNDFFHSFLI  937 
SCN9A-Mmu    867 FIFAVVGMQLFGKSYKECVCKINDDCTLPRWHMNDFFHSFLI  908 
SCN9A-Cf     883 FIFAVVGMQLFGKSYKECVCKINEDCTLPRWHMNDFFHSFLI  924 
SCN9A-Bt     877 FIFAVVGMQLFGKSYKECVCKINEDCTLPRWHMNDFFHSFLI  918 
Scn9-Mm      868 FIFAVVGMQLFGKSYKECVCKINENCKLPRWHMNDFFHSFLI  909 
Scn9a-Rn     877 FIFAVVGMQLFGKSYKECVCKINVDCKLPRWHMNDFFHSFLI  918 
Scn9a-Gg     893 FIFAVVGMQLFGKYYKECVCKISSDCELPRWHMHDFFHSFLI  934 
Scn2a-Xt     849 FIFAVVGMQLFGKSYKECVCKIAENCELPRWHMNDFFHSFLI  890 

cons         925 ************* ********  :* ******:********  966 

 **920 932**
SCN9A-Hs     909 VFRVLCGEWIETMWDCMEVAGQAMCLIVYMMVMVIGNLVVLN  950 
SCN9A-Pt     938 VFRVLCGEWIETMWDCMEVAGQAMCLIVYMMVMVIGNLVVLN  979 
SCN9A-Mmu    909 VFRVLCGEWIETMWDCMEVAGQAMCLIVYMMVMVIGNLVVLN  950 
SCN9A-Cf     925 VFRVLCGEWIETMWDCMEVAGQAMCLIVYMMVMVIGNLVVLN  966 
SCN9A-Bt     919 VFRVLCGEWIETMWDCMEVAGQAMCLIVYMMVMVIGNLVVLN  960 
Scn9-Mm      910 VFRVLCGEWIETMWDCMEVAGQTMCLIVYMMVMVIGNLVVLN  951 
Scn9a-Rn     919 VFRVLCGEWIETMWDCMEVAGQTMCLIVYMMVMVIGNLVVLN  960 
Scn9a-Gg     935 VFRVLCGEWIETMWDCMEVAGQPMCLTVFMMVMVIGNLVVLN  976 
Scn2a-Xt     891 VFRVLCGEWIETMWDCMEVAGQSMCLLVFMLVMVIGNLVVLN  932 

cons         967 **********************.*** *:*:*********** 1008 


SCN9A-Hs     951 LFLALLLSSFSSDNLTAIEEDPDANNLQIAVTRIKKGI----  988 
SCN9A-Pt     980 LFLALLLSSFSSDNLTAIEEDPDANNLQIAVTRIKKGINYGI 1021 
SCN9A-Mmu    951 LFLALLLSSFSSDNLTAIEEDPDANNLQIAVTRIKKGI----  988 
SCN9A-Cf     967 LFLALLLSSFSSDNLTAIEEDTDANNLQIAVARIKKGV---- 1004 
SCN9A-Bt     961 LFLALLLSSFSSDNLTAIEEDTDANNLQIAVARIKKGI----  998 
Scn9-Mm      952 LFLALLLSSFSSDNLTAIEEDTDANNLQIAVARIKRGI----  989 
Scn9a-Rn     961 LFLALLLSSFSSDNLTAIEEDTDANNLQIAVARIKRGI----  998 
Scn9a-Gg     977 LFLALLLSSFSSDSLSPTEDDNEMNNLQIAVARIQKGI---- 1014 
Scn2a-Xt     933 LFLALLLSSFSSDNLSATDEDSEINNIQIAVGRIQRGV----  970 

cons        1009 *************.*:. ::* : **:**** **::*:     1050 


SCN9A-Hs     989 NYVKQTLREFILKAFSKKPKISREIRQAEDLNTKKENYISNH 1030 
SCN9A-Pt    1022 NYVKQILREFILKAFSKKPKISREIRQAEDLNTKKENYISNH 1063 
SCN9A-Mmu    989 NYVKQTLREFILKTFSKKPKISREIRQTEDLNTKKENYISNY 1030 
SCN9A-Cf    1005 NYVKQTLREFILKAFSKKPKISKDTRRAEDQNSKKENCISNR 1046 
SCN9A-Bt     999 KYVKQTLREFVLKAFSKKPKISKEIRQTEDLNCKKENYISNR 1040 
Scn9-Mm      990 NYVKQTLREFILKSFSKKPKGSKDTKRTADPNNKRENYISNR 1031 
Scn9a-Rn     999 NYVKQTLREFILKSFSKKPKGSKDTKRTADPNNKKENYISNR 1040 
Scn9a-Gg    1015 DYVKEKVGEYIQKSCWRKQMAANERTATDQLNDERHHCISNC 1056 
Scn2a-Xt     971 ASGKIILREFFEKLFLQKQKDINETKQLEETPGKNDTCVLGN 1012 

cons        1051    *  : *:. *   :*    .:     :   :..  : .  1092 


SCN9A-Hs    1031 TLAEMSKGHNFLKEKDKI-SGFGSSVDKHLMEDSDGQSFIHN 1071 
SCN9A-Pt    1064 TLAEMSKGHNFLKEKDKI-SGFGSSMDKHLMEDSDGQSFIHN 1104 
SCN9A-Mmu   1031 TLAEMSKGHNFLKEKDKI-SGFGSCVDKYLMEDSDGQSFIHN 1071 
SCN9A-Cf    1047 TLAEMNKDHNFHKEKEKI-SGFGSSMDKYLMEESDCQSFIHN 1087 
SCN9A-Bt    1041 TLAEMSKDHKFHKEKDKT-SGFGNSMDKYLMEESDGQSFIHN 1081 
Scn9-Mm     1032 TLAEISKDHNFLKEKDKI-SGFSSSLDKSFMDENDYQSFIHN 1072 
Scn9a-Rn    1041 TLAEMSKDHNFLKEKDRI-SGYGSSLDKSFMDENDYQSFIHN 1081 
Scn9a-Gg    1057 TVAEIKADTTYHKNENGT-AGVVG--------SSDYPLFINN 1089 
Scn2a-Xt    1013 AVVEILKEKNDVRDSDGMTSEIGSSVEKSAENEKEYMTVINK 1054 

cons        1093 ::.*:    .  ::.:   :   .        ..:   .*:: 1134 


SCN9A-Hs    1072 PSLTVTVPIAPGESDLENMNAEELSSDSDSEYSKVRLNRSSS 1113 
SCN9A-Pt    1105 PSLTVTVPIAPGESDLENMNAEELSSDSDSEYSKVRLNQSSS 1146 
SCN9A-Mmu   1072 PSLTVTVPIAPGESDLENMNTEELSSDSDSEYSKVRLNQSSS 1113 
SCN9A-Cf    1088 PSLTVTVPIAPGESDLENMNTEELSSDSDSEYSKGRLNRSSS 1129 
SCN9A-Bt    1082 PSLTVTVPIAPGESDLEIMNTEELSSDSDSEYSKGRLNQSSS 1123 
Scn9-Mm     1073 PSLTVTVPIAPGESDLENMNTEELSSDSDSDYSKERRNRSSS 1114 
Scn9a-Rn    1082 PSLTVTVPIAPGESDLEIMNTEELSSDSDSDYSKEKRNRSSS 1123 
Scn9a-Gg    1090 PSLTVTVPIAVGESDFEHLNTEEFSSDSDLEESKEKINLSSS 1131 
Scn2a-Xt    1055 TSLTVAVPIAPGESDFENPNTEEFSSESDPEETKEKIKVSSS 1096 

cons        1135 .****:**** ****:*  *:**:**:** : :* : : *** 1176


SCN9A-Hs    1114 SECSTVDNPLPGEGEE----AEAEPMNSDEPEACFTDGCVRR 1151 
SCN9A-Pt    1147 SECSTVDNPLPGEGEE----AEAEPMNSDEPEACFTDGCVRR 1184 
SCN9A-Mm    1114 SECSTVDNPLPGEGEE----AEAEPMNSDEPEACFTDGCVRR 1151 
SCN9A-Cf    1130 SECSTVDNPLPGEGEE----AEAEPVNSDEPEACFTDGCVRR 1167 
SCN9A-Bt    1124 SECSTVDNPVPGEGEE----AEAEPVNSDEPEACFTDGCVQR 1161 
Scn9-Mm     1115 SECSTVDNPLPGE-EE----AEAEPINADEPEACFTDGCVRR 1151 
Scn9a-Rn    1124 SECSTVDNPLPGE-EE----AEAEPVNADEPEACFTDGCVRR 1160 
Scn9a-Gg    1132 SEGSTVNLALFGE-EK----AETEPEKAAELQTCFTEGCIQK 1168 
Scn2a-Xt    1097 SEGSTIDIRAPGV-ERIEEFLDYEYEETFDPEPCFTDDCVAS 1137 

cons        1177 ** **::    *  *.     : *  :: : :.***:.*:   1218 

 **1176**
SCN9A-Hs    1152 FSCCQVNIESGKGKIWWNIRKTCYKIVEHSWFESFIVLMILL 1193 
SCN9A-Pt    1185 FSCCQVNIESGKGKIWWNIRKTCYKIVEHSWFESFIVLMILL 1226 
SCN9A-Mm    1152 FSCCQVNIESGKGKIWWNIRKTCYKIVEHSWFESFIVLMILL 1193 
SCN9A-Cf    1168 FPCCQVDIESGKGKIWWNIRKTCYRIVEHSWFESFIVLMILL 1209 
SCN9A-Bt    1162 FPCCQVNIESGKGKIWWNIRKTCFRIVEHSWFESFIVLMILL 1203 
Scn9-Mm     1152 FPCCQVNIDSGKGKVWWTIRKTCYRIVEHSWFESFIVLMILL 1193 
Scn9a-Rn    1161 FPCCQVNVDSGKGKVWWTIRKTCYRIVEHSWFESFIVLMILL 1202 
Scn9a-Gg    1169 FKCCRCSIESRRGIIWWNLRKTCYRIVEHNWFETFIVFMILL 1210 
Scn2a-Xt    1138 FTCCQINIENGTGRDWWNLRKTCYTIVEHSWFESFIIFMILL 1179 

cons        1219 * **: .::.  *  **.:****: ****.***:**::**** 1260 

 **1207 1210 1235**
SCN9A-Hs    1194 SSGALAFEDIYIERKKTIKIILEYADKIFTYIFILEMLLKWI 1235 
SCN9A-Pt    1227 SSGALAFEDIYIERKKTIKIILEYADKIFTYIFILEMLLKWI 1268 
SCN9A-Mmu   1194 SSGALAFEDIYIERKKTIKIILEYADKIFTYIFILEMLLKWI 1235 
SCN9A-Cf    1210 SSGALAFEDIYIEKKKTIKIILEYADKIFTYIFILEMLLKWV 1251 
SCN9A-Bt    1204 SSGALAFEDIYIEKKKNIKIILEYADKIFTYIFILEMLLKWV 1245 
Scn9-Mm     1194 SSGALAFEDIYIEKKKTIKIILEYADKIFTYIFILEMLLKWV 1235 
Scn9a-Rn    1203 SSGALAFEDIYIEKKKTIKIILEYADKIFTYIFILEMLLKWV 1244 
Scn9a-Gg    1211 SSGTLAFEDIYIEQRKTIKVILDYADKIFTYIFILEMVLKWV 1252 
Scn2a-Xt    1180 SSGALAFEDIYVEQRRNVKAILEYADKVFAYIFIMEMLLKWV 1221 

cons        1261 ***:*******:*:::.:* **:****:*:****:**:***: 1302 

 **1245 1267**
SCN9A-Hs    1236 AYGYKTYFTNAWCWLDFLIVDVSLVTLVANTLGYSDLGPIKS 1277 
SCN9A-Pt    1269 AYGYKTYFTNAWCWLDFLIVDVSLVTLVANTLGYSDLGPIKS 1310 
SCN9A-Mmu   1236 AYGYKTYFTNAWCWLDFLIVDVSLVTLVANTLGYSDLGPIKS 1277 
SCN9A-Cf    1252 AYGYKTYFTNAWCWLDFLIVDVSLVTLVANTLGYSDLGPIKS 1293 
SCN9A-Bt    1246 AYGYKTYFTNAWCWLDFLIVDVSLVTLVANTLGYSDLGPIKS 1287 
Scn9-Mm     1236 AYGYKTYFTNAWCWLDFLIVDVSLVTLVANTLGYSDLGPIKS 1277 
Scn9a-Rn    1245 AYGYKTYFTNAWCWLDFLIVDVSLVTLVANTLGYSDLGPIKS 1286 
Scn9a-Gg    1253 AYGFQTYFTNAWCWLDFLIVDVSLVSLVATALGFSELGAIKS 1294 
Scn2a-Xt    1222 AYGFIKYFTNAWCWLDFIIVDISLVSLIANALGYSELGAIKS 1263 

cons        1303 ***: .***********:***:***:*:*.:**:*:**.*** 1344 

 **1298-9 1308 1316**
SCN9A-Hs    1278 LRTLRALRPLRALSRFEGMRVVVNALIGAIPSIMNVLLVCLI 1319 
SCN9A-Pt    1311 LRTLRALRPLRALSRFEGMRVVVNALIGAIPSIMNVLLVCLI 1352 
SCN9A-Mmu   1278 LRTLRALRPLRALSRFEGMRVVVNALIGAIPSIMNVLLVCLI 1319 
SCN9A-Cf    1294 LRTLRALRPLRALSRFEGMRVVVNALIGAIPSIMNVLLVCLI 1335 
SCN9A-Bt    1288 LRTLRALRPLRALSRFEGMRVVVNALIGAIPSIMNVLLVCLI 1329 
Scn9-Mm     1278 LRTLRALRPLRALSRFEGMRVVVNALIGAIPSIMNVLLVCLI 1319 
Scn9a-Rn    1287 LRTLRALRPLRALSRFEGMRVVVNALIGAIPSIMNVLLVCLI 1328 
Scn9a-Gg    1295 LRTLRALRPLRALSRFEGMRVVVNALTGAIPSIMNVLLVCLT 1336 
Scn2a-Xt    1264 LRTLRALRPLRALSRFEGMRVVVNALIGAIPSIMNVLLVCLI 1305 

cons        1345 ************************** **************  1386 


SCN9A-Hs    1320 FWLIFSIMGVNLFAGKFYECINTTDGSRFPASQVPNRSECFA 1361 
SCN9A-Pt    1353 FWLIFSIMGVNLFAGKFYECINTTDGSRFPASQVPNRSECFA 1394 
SCN9A-Mmu   1320 FWLIFSIMGVNLFAGKFYECINTTDGSRFPASQVPNRSECFA 1361 
SCN9A-Cf    1336 FWLIFSIMGVNLFAGKFYECVNTTDGSRFPTNLVQNHSDCFA 1377 
SCN9A-Bt    1330 FWLIFSIMGVNLFAGKFYECINTTNGLRFPTSEVENRSACLA 1371 
Scn9-Mm     1320 FWLIFSIMGVNLFAGKFYECVNTTDGSRFSVSQVANRSECFA 1361 
Scn9a-Rn    1329 FWLIFSIMGVNLFAGKFYECVNTTDGSRFPTSQVANRSECFA 1370 
Scn9a-Gg    1337 FWLIFSIMGVNLFAGKFFYCVNTTTGVQFKPYEVDNKSMCEN 1378 
Scn2a-Xt    1306 FWLIFSIMGVNLFAGKFYYCVNTTSGVPFPATDVNNYSDCRM 1347 

cons        1387 *****************: *:*** *  *    * * * *   1428 

 **1398-9**
SCN9A-Hs    1362 LMNVSQNVRWKNLKVNFDNVGLGYLSLLQVATFKGWTIIMYA 1403 
SCN9A-Pt    1395 LMNVSQNVRWKNLKVNFDNVGLGYLSLLQVATFKGWTIIMYA 1436 
SCN9A-Mmu   1362 LMNVSQNVRWKNLKVNFDNVGLGYLSLLQVATFKGWTIIMYA 1403 
SCN9A-Cf    1378 LMNVSQNVRWKNLKVNFDNVGLGYLSLLQVATFKGWMDIMYA 1419 
SCN9A-Bt    1372 LMNVSQNVRWKNLKVNFDNVGLGYLSLLQVATFKGWMDIMYA 1413 
Scn9-Mm     1362 LMNVSGNVRWKNLKVNFDNVGLGYLSLLQVATFKGWMDIMYA 1403 
Scn9a-Rn    1371 LMNVSGNVRWKNLKVNFDNVGLGYLSLLQVATFKGWMDIMYA 1412 
Scn9a-Gg    1379 LGNTASDVRWKNVKVNFDNVGAGYLSLLQVATFKGWMEIMYA 1420 
Scn2a-Xt    1348 YINTTGAARWKNVKVNFDNVGAGYLALLQVATFKGWMPIMYA 1389 

cons        1429   *.:  .****:******** ***:**********  **** 1470 

 **1411-2 1415 1419 1428**
SCN9A-Hs    1404 AVDSVNVDKQPKYEYSLYMYIYFVVFIIFGSFFTLNLFIGVI 1445 
SCN9A-Pt    1437 AVDSVNVDKQPKYEYSLYMYIYFVVFIIFGSFFTLNLFIGVI 1478 
SCN9A-Mm    1404 AVDSVNVDKQPKYEYSLYMYIYFVIFIIFGSFFTLNLFIGVI 1445 
SCN9A-Cf    1420 AVDSVNVSIQPIYEYNLYMYIYFVIFIIFGSFFTLNLFIGVI 1461 
SCN9A-Bt    1414 AVDSVNVNKQPIYEYSLYMYIYFVIFIIFGSFFTLNLFIGVI 1455 
Scn9-Mm     1404 AVDSVNVNAQPIYEYNLYMYIYFVIFIIFGSFFTLNLFIGVI 1445 
Scn9a-Rn    1413 AVDSVNVNEQPKYEYSLYMYIYFVIFIIFGSFFTLNLFIGVI 1454 
Scn9a-Gg    1421 AVDSRDVGKQPMYEDNLYMYLYFVAFIIFGSFFTLNLFIGVI 1462 
Scn2a-Xt    1390 AVDSREINEQPKYENNTTMYLYFVAFIIFGSFFTLNLFIGVI 1431 

cons        1471 **** ::. ** ** .  **:*** ***************** 1512 


SCN9A-Hs    1446 IDNFNQQKKKLGGQDIFMTEEQKKYYNAMKKLGSKKPQKPIP 1487 
SCN9A-Pt    1479 IDNFNQQKKKLGGQDIFMTEEQKKYYNAMKKLGSKKPQKPIP 1520 
SCN9A-Mm    1446 IDNFNQQKKKLGGQDIFMTEEQKKYYNAMKKLGSKKPQKPIP 1487 
SCN9A-Cf    1462 IDNFNQQKKKLGGQDIFMTEEQKKYYNAMKKLGSKKPQKPIP 1503 
SCN9A-Bt    1456 IDNFNQQKKKLGGQDIFMTEEQKKYYNAMKKLGSKKPQKPIP 1497 
Scn9-Mm     1446 IDNFNQQKKKLGGQDIFMTEEQKKYYNAMKKLGSKKPQKPIP 1487 
Scn9a-Rn    1455 IDNFNQQKKKLGGQDIFMTEEQKKYYNAMKKLGSKKPQKPIP 1496 
Scn9a-Gg    1463 IDNFNQQKKKLGGQDIFMTEEQKKYYNAMKKLGSKKPQKPIP 1504 
Scn2a-Xt    1432 IDNFNQQKKKIRGQDIFMTEEQKKYYNAMKKLGSKKPQKPIP 1473 

cons        1513 **********: ****************************** 1554 

 **1505 1509**
SCN9A-Hs    1488 RPGNKIQGCIFDLVTNQAFDISIMVLICLNMVTMMVEKEGQS 1529 
SCN9A-Pt    1521 RPGNKIQGCIFDLVTNQAFDISIMVLICLNMVTMMVEKEGQS 1562 
SCN9A-Mmu   1488 RPGNKIQGCIFDLVTNQAFDISIMVLICLNMVTMMVEKEGQS 1529 
SCN9A-Cf    1504 RPGNKFQGCIFDLVTNQVFDITIMVLICLNMVTMMVEKEGQS 1545 
SCN9A-Bt    1498 RPGNKFQGCIFDLVTNQAFDIAIMVLICLNMVTMMVEKEGQS 1539 
Scn9-Mm     1488 RPGNKFQGCIFDLVTNQAFDITIMVLICLNMVTMMVEKEGQT 1529 
Scn9a-Rn    1497 RPGNKFQGCIFDLVTNQAFDITIMVLICLNMVTMMVEKEGQT 1538 
Scn9a-Gg    1505 RPANKLQGLVFDIVTKQAFDIGIMVLICLNMVTMMIETDDQS 1546 
Scn2a-Xt    1474 RPANCCQGLIFDFVTKQAFDIIIMILIFLNMVTMMIETDDQS 1515 

cons        1555 **.*  ** :**:**:*.*** **:** *******:*.:.*: 1596 

 **1530-2** **1534 1537-8 1548 1560 1565**
SCN9A-Hs    1530 QHMTEVLYWINVVFIILFTGECVLKLISLRHYYFTVGWNIFD 1571 
SCN9A-Pt    1563 QHMTEVLYWINVVFIILFTGECVLKLISLRHYYFTVGWNIFD 1604 
SCN9A-Mmu   1530 PYMTDVLYWINVVFIILFTGECVLKLISLRYYYFTIGWNIFD 1571 
SCN9A-Cf    1546 KYMTDVLYWINVVFIILFTGECVLKLISLRHYYFTVGWNIFD 1587 
SCN9A-Bt    1540 DYVTEVLNWINVVFIILFSGECVLKLISLRCYYFTVGWNIFD 1581 
Scn9-Mm     1530 DYMSFVLYWINVVFIILFTGECVLKLISLRHYYFTVGWNIFD 1571 
Scn9a-Rn    1539 EYMDYVLHWINMVFIILFTGECVLKLISLRHYYFTVGWNIFD 1580 
Scn9a-Gg    1547 ELMQNILYWINFVFVVLFTGECVLKLFSLRYYYFTVGWNIFD 1588 
Scn2a-Xt    1516 QEMEMYLYRINAVFIILFTGEFLLKLISLRQYYFTIGWNIFD 1557 

cons        1597   :   *  ** **::**:** :***:*** ****:****** 1638

 **1577 1586 1590 1607 1613**
SCN9A-Hs    1572 FVVVIISIVGMFLADLIETYFVSPTLFRVIRLARIGRILRLV 1613 
SCN9A-Pt    1605 FVVVIISIVGMFLADLIETYFVSPTLFRVIRLARIGRILRLV 1646 
SCN9A-Mmu   1572 FVVVIISIVGMFLADLIETYFVSPTLFRVIRLARIGRILRLV 1613 
SCN9A-Cf    1588 FVVVILSIVGMFLAELIEKYFVSPTLFRVIRLARIGRILRLI 1629 
SCN9A-Bt    1582 FVVVILSIVGMFLADLIERYFVSPTLFRVIRLARIGRILRLI 1623 
Scn9-Mm     1572 FVVVILSIVGMFLAEMIEKYFVSPTLFRVIRLARIGRILRLI 1613 
Scn9a-Rn    1581 FVVVILSIVGMFLAEMIEKYFVSPTLFRVIRLARIGRILRLI 1622 
Scn9a-Gg    1589 FVVVILSIVGMFLAKVIEKYFVSPTLFRVIRLARIGRILRLI 1630 
Scn2a-Xt    1558 LVVVILSIVGMFLADIIERYFFSPTLFRVIRLARIGRILRLI 1599 

cons        1639 :****:********.:** **.*******************: 1680

 **1627 1632**
SCN9A-Hs    1614 KGAKGIRTLLFALMMSLPALFNIGLLLFLVMFIYAIFGMSNF 1655 
SCN9A-Pt    1647 KGAKGIRTLLFALMMSLPALFNIGLLLFLVMFIYAIFGMSNF 1688 
SCN9A-Mmu   1614 KGAKGIRTLLFALMMSLPALFNIGLLLFLVMFIYAIFGMSNF 1655 
SCN9A-Cf    1630 KGAKGIRTLLFALMMSLPALFNIGLLLFLVMFIYAIFGMSNF 1671 
SCN9A-Bt    1624 KGAKGIRTLLFALMMSLPALFNIGLLLFLVMFIYAIFGMSNF 1665 
Scn9-Mm     1614 KGAKGIRTLLFALMMSLPALFNIGLLLFLVMFIYAIFGMSNF 1655 
Scn9a-Rn    1623 KGAKGIRTLLFALMMSLPALFNIGLLLFLVMFIYAIFGMSNF 1664
Scn9a-Gg    1631 KGAKGIRTLLFALMMSLPALFNIGLLLFLVMFIYAIFGMSNF 1672 
Scn2a-Xt    1600 KAAKGIRTLLFALMMSLPALFNIGLLLFLVMFIYAIFGMSNF 1641 

cons        1681 *.**************************************** 1722

 **1662 1674**
SCN9A-Hs    1656 AYVKKEDGINDMFNFETFGNSMICLFQITTSAGWDGLLAPIL 1697 
SCN9A-Pt    1689 AYVKKEDGINDMFNFETFGNSMICLFQITTSAGWDGLLAPIL 1730 
SCN9A-Mmu   1656 AYVKKEDGINDMFNFETFGNSMICLFQITTSAGWDGLLAPIL 1697 
SCN9A-Cf    1672 AYVKKEAGINDMFNFETFGNSMICLFQITTSAGWDGLLAPIL 1713 
SCN9A-Bt    1666 AYVKKEAGINDMFNFETFANSMICLFQITTSAGWDGLLAPIL 1707 
Scn9-Mm     1656 AYVKKEAGINDMFNFETFGNSMICLFQITTSAGWDGLLAPIL 1697 
Scn9a-Rn    1665 AYVKKEAGINDMFNFETFGNSMICLFQITTSAGWDGLLAPIL 1706 
Scn9a-Gg    1673 AYVKREAGIDDMFNFETFGNSMICLFQITTSAGWDGLLAPIL 1714 
Scn2a-Xt    1642 AYVKKQSGIDDMFNFETFGNSMICLFQITTSAGWDLLLEPIL 1683 

cons        1723 ****:: **:********.**************** ** *** 1764

 **1700**
SCN9A-Hs    1698 NSKPPDCDPKKVHPGSSVEGDCGNPSVGIFYFVSYIIISFLV 1739 
SCN9A-Pt    1731 NSKPPDCDPKKVHPGSSVEGDCGNPSVGIFYFVSYIIISFLV 1772 
SCN9A-Mmu   1698 NSKPPDCDPKKVHPGSSVEGDCGNPSVGIFYFVSYIIISFLV 1739 
SCN9A-Cf    1714 NSAPPDCDPKKVHPGSSVEGDCGNPSVGIFYFVSYIIISFLV 1755 
SCN9A-Bt    1708 NSKPPDCDPKKVHPGSSVEGDCGNPSVGIFYFVSYIIISFLV 1749 
Scn9-Mm     1698 NSAPPDCDPKKVHPGSSVEGDCGNPSVGIFYFVSYIIISFLV 1739 
Scn9a-Rn    1707 NSAPPDCDPKKVHPGSSVEGDCGNPSVGIFYFVSYIIISFLV 1748 
Scn9a-Gg    1715 NSGKPDCDPNKPHPGSSVKGDCGNPSVGIFFFVSYIIISFLV 1756 
Scn2a-Xt    1684 NSGEPDCDPNAEHPGSLFKGDCGNPSVGIFFFVSYIIISFLI 1725

cons        1765 **  *****:  **** .:***********:**********: 1806 

 **1746**
SCN9A-Hs    1740 VVNMYIAVILENFSVATEESTEPLSEDDFEMFYEVWEKFDPD 1781 
SCN9A-Pt    1773 VVNMYIAVILENFSVATEESTEPLSEDDFEMFYEVWEKFDPD 1814 
SCN9A-Mmu   1740 VVNMYIAVILENFSVATEESTEPLSEDDFEMFYEVWEKFDPD 1781 
SCN9A-Cf    1756 VVNMYIAVILENFSVATEESTEPLSEDDFEMFYEVWEKFDPD 1797 
SCN9A-Bt    1750 VVNMYIAVILENFSVATEESTEPLSEDDFEMFYEVWEKFDPD 1791 
Scn9-Mm     1740 VVNMYIAVILENFSVATEESTEPLSEDDFEMFYEVWEKFDPD 1781 
Scn9a-Rn    1749 VVNMYIAVILENFSVATEESTEPLSEDDFEMFYEVWEKFDPD 1790 
Scn9a-Gg    1757 VVNMYIAVILENFGVATEESAEPLSEDDFEMFYEVWEKYDPD 1798 
Scn2a-Xt    1726 VVNMYIAVILENFSVATEESAEPLGEDDFEMFYEVWEKFDPS 1767 

cons        1807 *************.******:***.*************:**. 1848


SCN9A-Hs    1782 ATQFIEFSKLSDFAAALDPPLLIAKPNKVQLIAMDLPMVSGD 1823 
SCN9A-Pt    1815 ATQFIEFSKLSDFAAALDPPLLIAKPNKVQLIAMDLPMVSGD 1856 
SCN9A-Mmu   1782 ATQFIEYNKLSDFAAALDPPLLIAKPNKVQLIAMDLPMVSGD 1823 
SCN9A-Cf    1798 ATQFIEYSKLSDFAAALDPPLLIAKPNKVQLIAMDLPMVSGD 1839 
SCN9A-Bt    1792 ATQFIEYSKLSDFAAALDPPLLIAKPNKVQLIAMDLPMVSGD 1833 
Scn9-Mm     1782 ATQFIEFCKLSDFAAALDPPLLIAKPNKVQLIAMDLPMVSGD 1823 
Scn9a-Rn    1791 ATQFIEFCKLSDFAAALDPPLLIAKPNKVQLIAMDLPMVSGD 1832 
Scn9a-Gg    1799 ATQFIEYSKLSDFAASLDPPLNIPKPNKVQLIAMDLPMVSGD 1840 
Scn2a-Xt    1768 ASQFIEYSKLSDFADALDPPLRVPKPNNIQLIAMDLPMVSGD 1809 

cons        1849 *:****: ****** :***** :.***::************* 1890


SCN9A-Hs    1824 RIHCLDILFAFTKRVLGESGEMDSLRSQMEERFMSANPSKVS 1865 
SCN9A-Pt    1857 RIHCLDILFAFTKRVLGESGEMDSLRSQMEERFMSANPSKVS 1898 
SCN9A-Mmu   1824 RIHCLDILFAFTKRVLGESGEMDSLRSQMEERFMSANPSKVS 1865 
SCN9A-Cf    1840 RIHCLDILFAFTKRVLGESGEMDSLRSQMEERFMSANPSKVS 1881 
SCN9A-Bt    1834 RIHCLDILFAFTKRVLGEGGEMDSLRSQMEERFMSANPSKVS 1875 
Scn9-Mm     1824 RIHCLDILFAFTKRVLGESGEMDSLRSQMEERFMSANPSKVS 1865 
Scn9a-Rn    1833 RIHCLDILFAFTKRVLGEGGEMDSLRSQMEERFMSANPSKVS 1874 
Scn9a-Gg    1841 RIHCLDILFAFTKRVLGESDEMDALRVQMEDRFMAANPSKVS 1882 
Scn2a-Xt    1810 RIHCLDILFAFTKRVLGEGGEMDSLRQPMEERFMTSNPSKVP 1851 

cons        1891 ******************..***:**  **:***::*****. 1932 


SCN9A-Hs    1866 YEPITTTLKRKQEDVSATVIQRAYRRYRLRQNVKNISSIYIK 1907 
SCN9A-Pt    1899 YEPITTTLKRKQEDVSATVIQRAYRRYRLRQNVKNISSIYIK 1940 
SCN9A-Mmu   1866 YEPITTTLKRKQEDVSATVIQRAYRRYRLRQNVKNISSIYIK 1907 
SCN9A-Cf    1882 YEPITTTLKRKQEDVSATVIQRAYRRYRLRQNVKNISSIYIK 1923 
SCN9A-Bt    1876 YEPITTTLKRKQEDVSATVIQRAYRRYRLRQNVKNISSIYIK 1917 
Scn9-Mm     1866 YEPITTTLKRKQEDVSATIIQRAYRRYRLRQNVKNISSIYIK 1907 
Scn9a-Rn    1875 YEPITTTLKRKQEEVSATIIQRAYRRYRLRQHVKNISSIYIK 1916 
Scn9a-Gg    1883 YEPITTTLKRKQEEVSATIIQRAYRCYLLRRSIKKLSCMYRK 1924 
Scn2a-Xt    1852 YEPITTTLRRKQEEQSAVVIQRCYRRFRLKKR--QASQTY-- 1889 

cons        1933 ********:****: **.:***.** : *::   : *  *   1974


SCN9A-Hs    1908 DGDRDDDLLNKKDMAFDNVNENSSPEKTDATSSTTSPPSYDS 1949 
SCN9A-Pt    1941 DGDRDDDLLNKKDMAFDNVNENSSPEKTDATSSTTSPPSYDS 1982 
SCN9A-Mmu   1908 DGDRDDDLLNKKDMAFDNVNENSSPEKTDATSSTTSPPSYDS 1949 
SCN9A-Cf    1924 DGDRDDDLPNKEDMVFDNI-ENSSPEKTDATPSTVSPPSYDS 1964 
SCN9A-Bt    1918 DGDRDDDLPNKEDMVFDNVNENSSPEKTGATPSTVSPPSYDS 1959 
Scn9-Mm     1908 DGDRDDDLPNKEDIVFDNVNENSSPEKTDATASTISPPSYDS 1949 
Scn9a-Rn    1917 DGDRDDDLPNKEDTVFDNVNENSSPEKTDVTASTISPPSYDS 1958 
Scn9a-Gg    1925 DGV---DVLSKNYMLFGKLSENSASEKTNMTASTTYPPSYDS 1963 
Scn2a-Xt    1890 KGNRNKQIEEIERNV-NKSQQRYTTEISDTSTSTMSPPSYDS 1930 

cons        1975 .*    :: . :    .:  :. :.* :. :.**  ****** 2016

SCN9A-Hs    1950 VTKPDKEKYEQDRTEKEDKGKDSKESKK 1977 
SCN9A-Pt    1983 VTKPDKEKYEQDRTEKEDKGKDSKESKK 2010 
SCN9A-Mmu   1950 VTKPDKEKYEQDRTEKEDKGKDSKESKK 1977 
SCN9A-Cf    1965 VTKPDKEKYEKDKTEKEDKGKDGKESKK 1992 
SCN9A-Bt    1960 VTKPDREKYEKDKTEKEDKGKDGKEGKK 1987 
Scn9-Mm     1950 VTKPDQEKYETDKTEKEDKEKD--ESRK 1975 
Scn9a-Rn    1959 VTKPDQEKYETDKTEKEDKEKD--ESRK 1984 
Scn9a-Gg    1964 VTKQEKEKYEDDKSEKEDKGKDRKGNKK 1991 
Scn2a-Xt    1931 VEKTQQEKYEKDRQKKKISLKTQNDV-H 1957 

cons        2017 * * ::**** *: :*: . *      : 2044
