# Supplementary material for: Network topology of NaV1.7 mutations in sodium channel-related painful disorders
Source: BMC Syst Biol. 2017 Feb 24;11:28. doi: 10.1186/s12918-016-0382-0 (PMC5324268; doi:10.1186/s12918-016-0382-0)
Supplement: Additional file 6: Figure S2. — Degree variation (∆D) in NaV1.7 mutations compared to WT. (DOCX 1691 kb) [file 12918_2016_382_MOESM6_ESM.docx]

**Figure S2** Degree variation (∆D) in NaV1.7 mutations compared to WT.


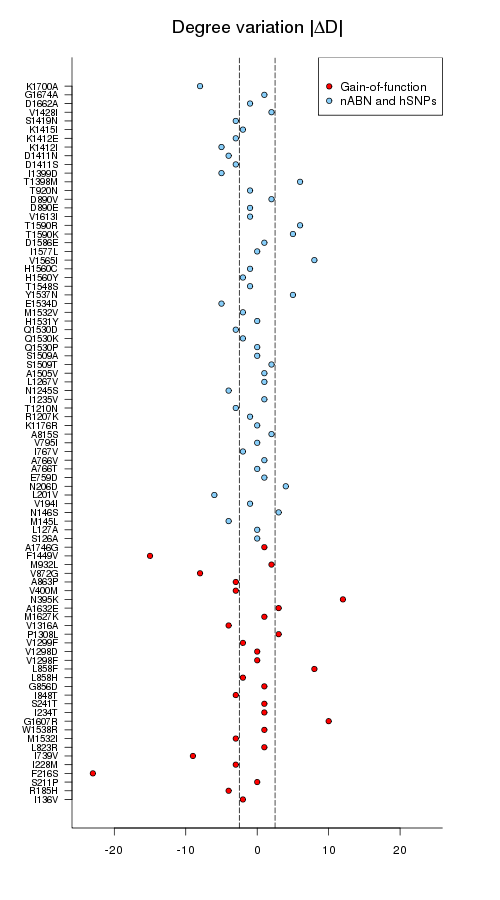


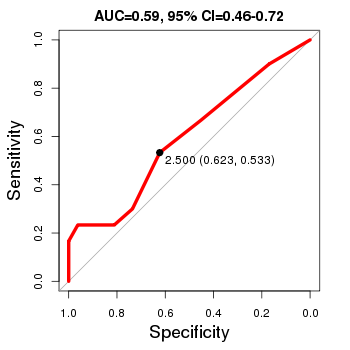


Left panel shows ∆D profile of WT and mutations. Positive and negative ∆D variations are found in mutations compared to Wt. Right panel shows Receiver Operating Curve (ROC) of gain-of-function and control (nABN and hSNPs) mutations as a function of ∆D using a cut-off of ± 2.5 (dashed lines). The area under the curve was 0.59 (95% Confidence Interval=0.46 to 0.72) sensitivity of 53% and specificity 62%.
