# Supplementary material for: Network topology of NaV1.7 mutations in sodium channel-related painful disorders
Source: BMC Syst Biol. 2017 Feb 24;11:28. doi: 10.1186/s12918-016-0382-0 (PMC5324268; doi:10.1186/s12918-016-0382-0)
Supplement: Additional file 7: Figure S3. — Clustering coefficient variation (∆CCct) in NaV1.7 mutations compared to WT. (DOCX 2798 kb) [file 12918_2016_382_MOESM7_ESM.docx]

**Figure S3** Clustering coefficient variation (∆CC*_ct_*) in NaV1.7 mutations.

*_
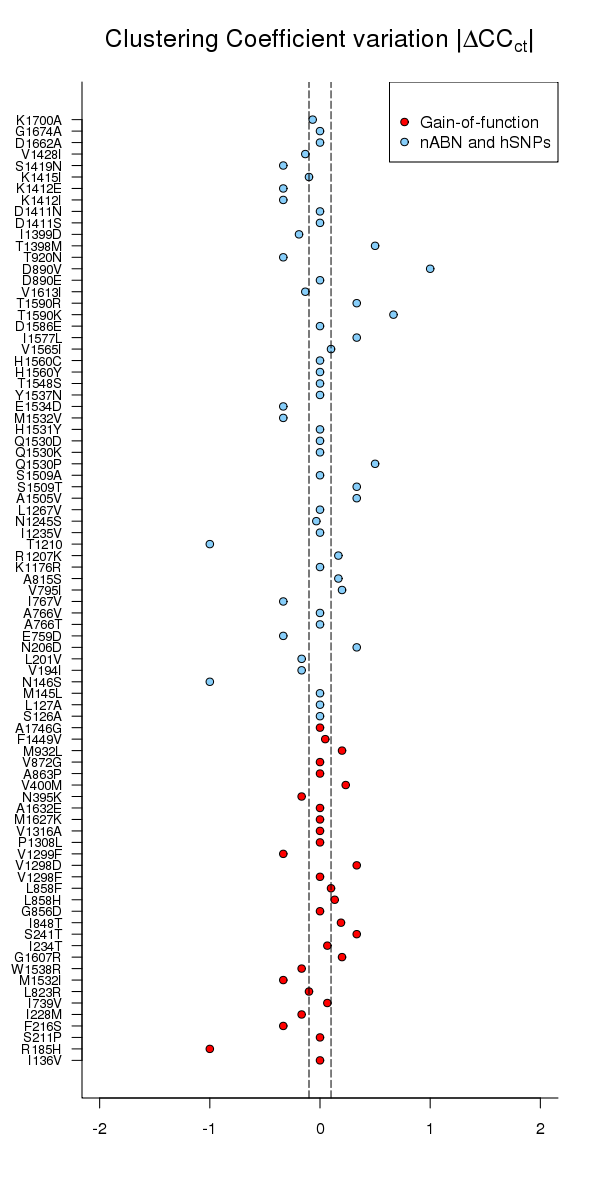
_*

*_
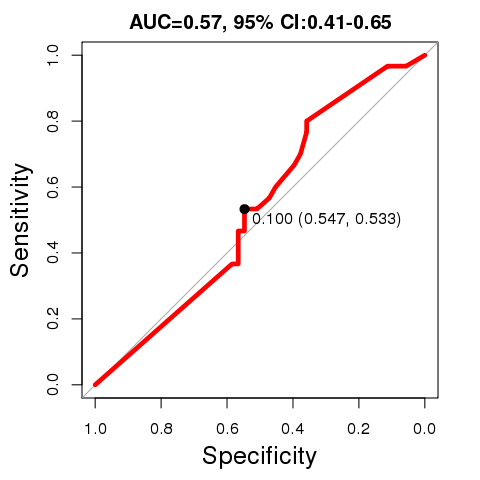
_*

Left panel shows ∆CC*_ct_* coefficient profile of WT and mutations. Positive and negative ∆CC*_ct_* variations are found in mutations compared to WT.

Right panel shows Receiver Operating Curve (ROC) of gain-of-function and control (nABN and hSNPs) mutations as a function of ∆CC*_ct_* using a cut-off of ± 0.10 (dashed lines). The area under the curve was 0.57 (95% Confidence Interval=0.41 to 0.65) with sensitivity of 54% and specificity 53%.
