# Supplementary material for: Network topology of NaV1.7 mutations in sodium channel-related painful disorders
Source: BMC Syst Biol. 2017 Feb 24;11:28. doi: 10.1186/s12918-016-0382-0 (PMC5324268; doi:10.1186/s12918-016-0382-0)
Supplement: Additional file 8: Figure S4. — Closeness Centrality variation (∆Cct) in NaV1.7 mutations compared to WT.A (DOCX 2801 kb) [file 12918_2016_382_MOESM8_ESM.docx]

**Figure S4** Closeness Centrality variation (∆C*_ct_*) in NaV1.7.

**A**


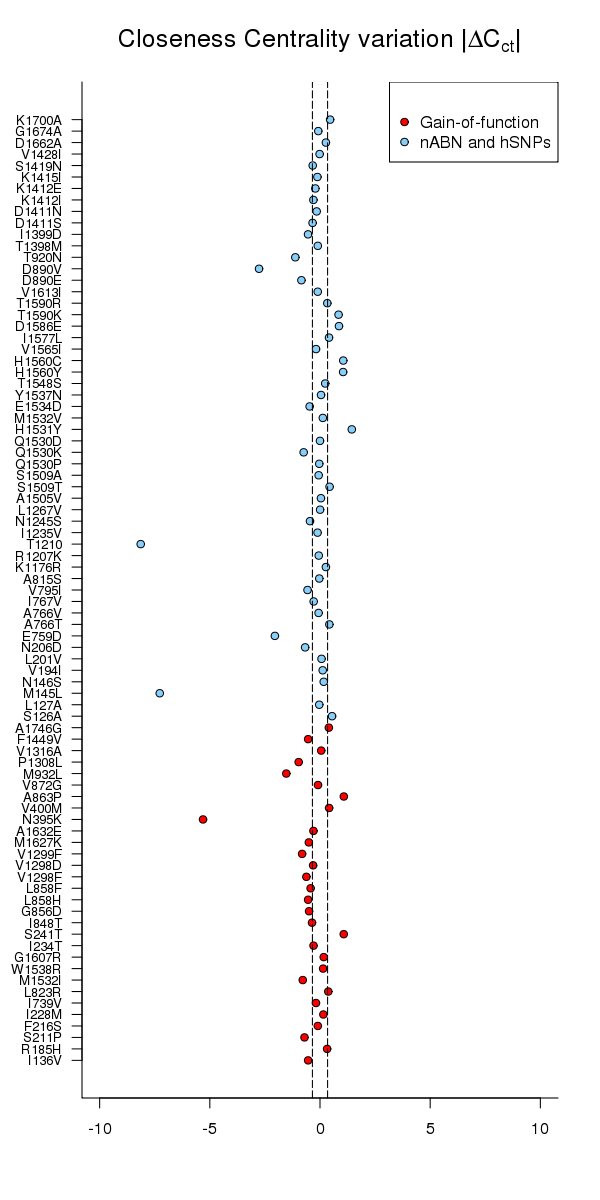


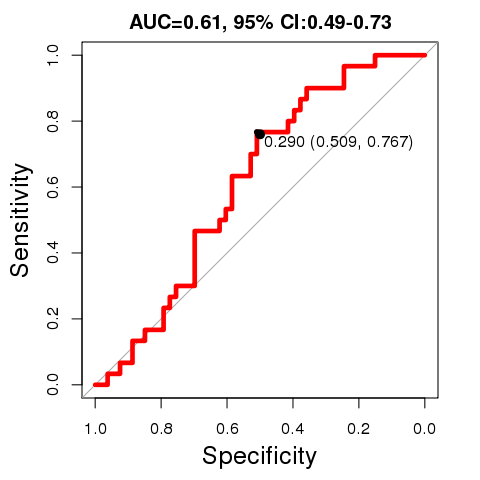


Left panel shows ∆C*_ct_* profile of WT and mutations. Positive and negative ∆C*_ct_* variations are found in mutations compared to WT. Right panel shows Receiver Operating Curve (ROC) of gain-of-function and control (nABN and hSNPs) mutations as a function of ∆C*_ct_* using a cut-off of ± 0.353 (dashed lines). The area under the curve was 0.61 (95% Confidence Interval=0.49 to 0.73) with sensitivity of 76% and specificity 50%.
