# Supplementary material for: Network topology of NaV1.7 mutations in sodium channel-related painful disorders
Source: BMC Syst Biol. 2017 Feb 24;11:28. doi: 10.1186/s12918-016-0382-0 (PMC5324268; doi:10.1186/s12918-016-0382-0)
Supplement: Additional file 9: Figure S5. — Eccentricity centrality variation (∆Ect) in NaV1.7 mutations compared to WT. (DOCX 2709 kb) [file 12918_2016_382_MOESM9_ESM.docx]

**Figure S5** Eccentricity centrality variation (∆E*_ct_*) in NaV1.7 mutations.


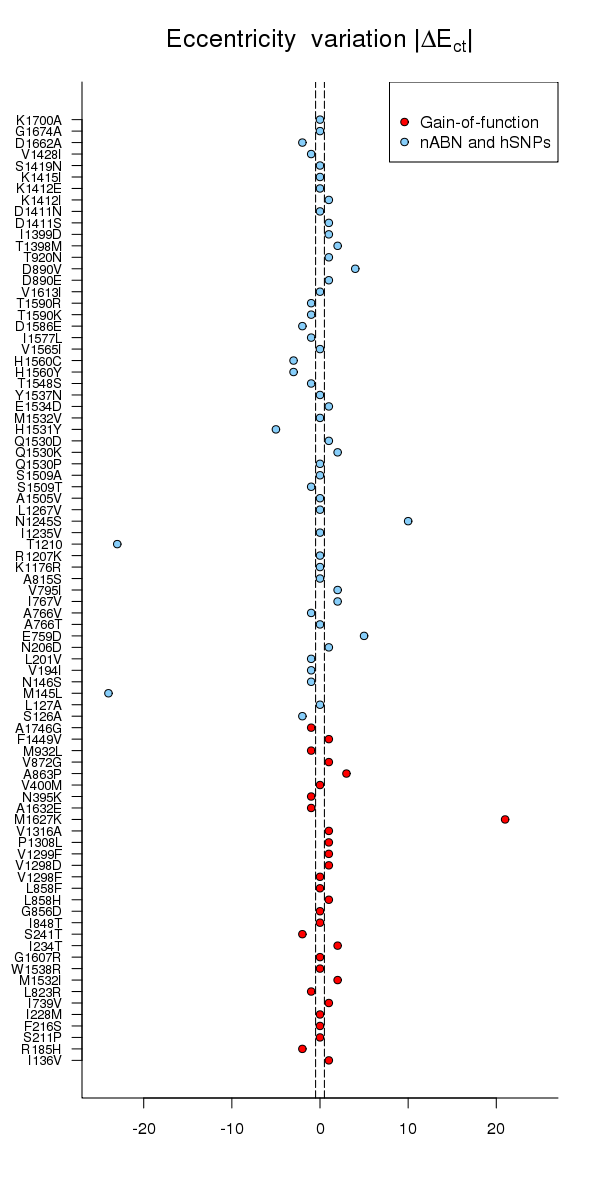


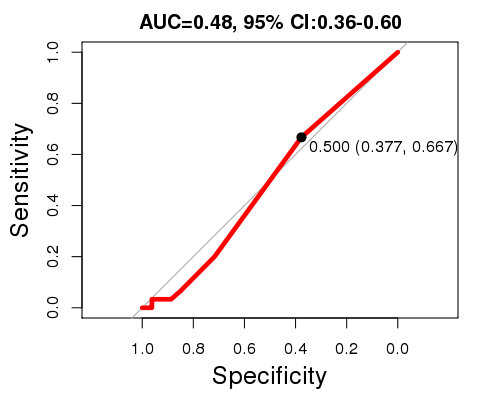


Left panel shows ∆E*_ct_* profile of WT and mutations. Positive and negative ∆E*_ct_* variations are found in mutations compared to WT.

Right panel shows Receiver Operating Curve (ROC) of gain-of-function and control (nABN and hSNPs) mutations as a function of ∆E*_ct_* using a cut-off of ± 0.5 (dashed lines). The area under the curve was 0.48 (95% Confidence Interval=0.36 to 0.60) sensitivity of 66% and specificity 37%.
