# Supplementary material for: Network topology of NaV1.7 mutations in sodium channel-related painful disorders
Source: BMC Syst Biol. 2017 Feb 24;11:28. doi: 10.1186/s12918-016-0382-0 (PMC5324268; doi:10.1186/s12918-016-0382-0)
Supplement: Additional file 16: Figure S6. — Structural modelling variants and their interatomic bonds of I848T and N395K. (DOCX 747 kb) [file 12918_2016_382_MOESM16_ESM.docx]

**Figure S6** Structural modelling variants and their interatomic bonds of I848T and N395K


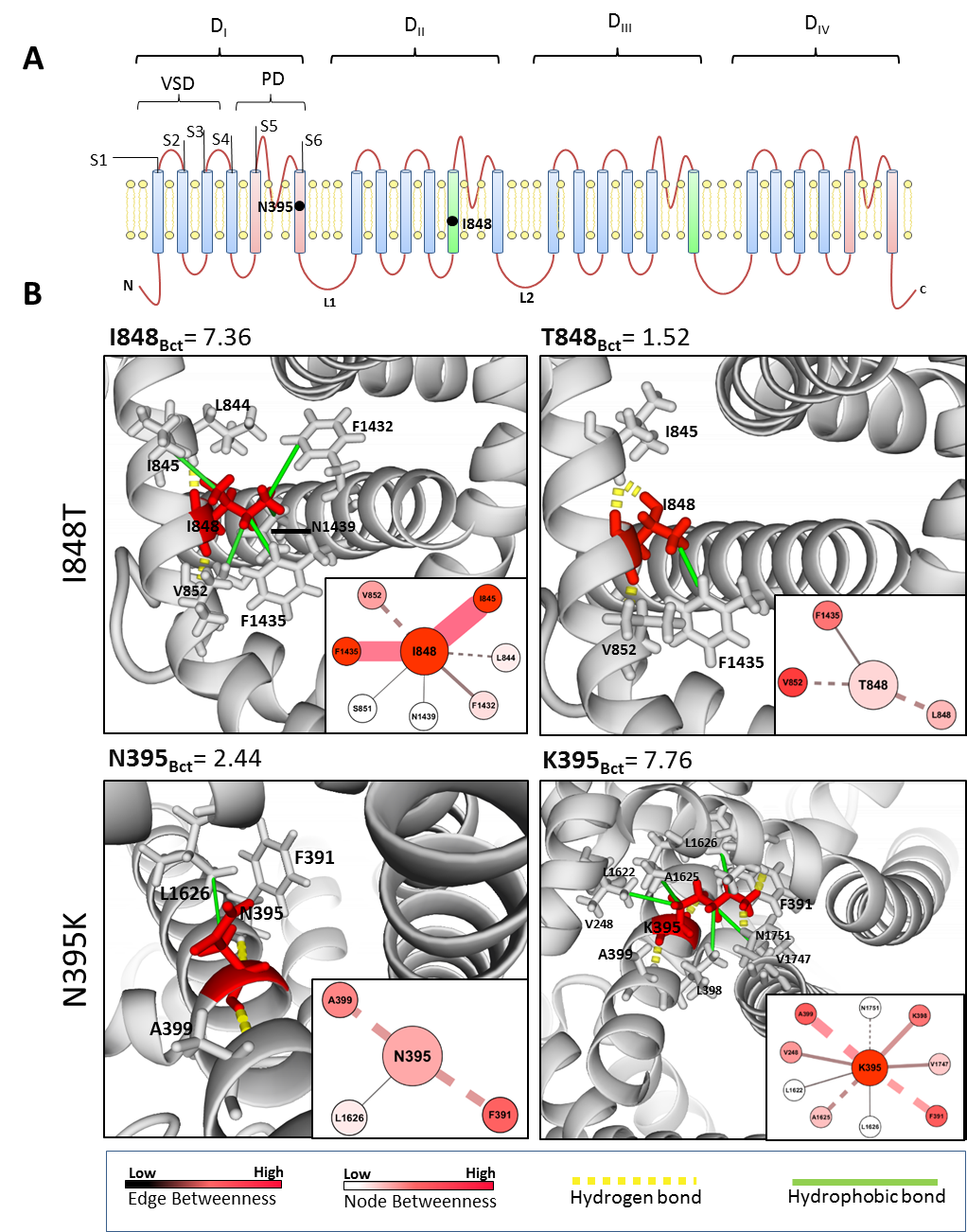


1. The graph shows the NaV1.7 sodium channel topology and highlights the amino acids I848 (D_I_; S4-S5) and N395 (D_I_; S6). Inter-domain bond interaction are depicted in red for the IEM associated mutation I848T and in green for the IEM associated mutation N395K.
2. Upper panels show interatomic bonds of I848 (left) and T848 (right). Lower panels show N395 (left) and K395 (right) interatomic bonds. Upper panels show that I848 interacts with S4-S5 (D_II_) and pore (D_III_) through I845 and F1435 that are two residues having very high B*_ct_* values (3.4 and 6.6, respectively). Note the difference of B_ct_ of the upper left panel (I848B_ct_=7.36) compared to the upper right panel (T848B_ct_=1.52). I848T mutation interrupts the shortest paths within the network between D_II_ (S4-S5) and D_III_ (pore) and therefore ΔB*_ct_* shifts to a negative value (-5.83). Hydrophobic bonds are showed in green solid lines. H-bonds are showed with yellow dashed lines. I848T mutation interacts through hydrophobic interactions (T848 and F1435) and H-bonds (T848[CO] with S851[NH]; T848[NH] with L844[CO]; T848[HG1] with L844[CO]). N395 amino acid (red, S6 in pore module in D_I_) interacts with L1626 (S4-S5) via hydrophobic bond and via H-bonds formed by N395[CO] and A399[NH] and N395[NH] with F391[CO]. K395 mutation creates new hydrophobic bonds with V248 (S4-S5, D_I_), L398 (S6, D_I_), V1747 (S6, D_IV_), L1622 (S4-S5, D_IV_) and new H-bonds formed by K395[NZ] with N1751[CG] (S6, D_IV_) and K395[NZ] with A1625[CO] (S4-S5, D_IV_). Red-to-white color gradient of amino acids (nodes) represents B_ct_ value (red represents high B_ct_ and white low B_ct_). Red-to-black color gradient of edges (amino acid interatomic interactions) corresponds to EB_ct_ value (red represents high EB_ct_ and black low EB_ct_).
